# GSK3β-mediated tau hyperphosphorylation triggers diabetic retinal neurodegeneration by disrupting synaptic and mitochondrial functions

**DOI:** 10.1186/s13024-018-0295-z

**Published:** 2018-11-22

**Authors:** Huazhang Zhu, Weizhen Zhang, Yingying Zhao, Xingsheng Shu, Wencong Wang, Dandan Wang, Yangfan Yang, Zhijun He, Xiaomei Wang, Ying Ying

**Affiliations:** 1 Department of Physiology, School of Basic Medical Sciences, Center for Diabetes, Obesity and Metabolism, Shenzhen University Health Sciences Center, Shenzhen, 518060 Guangdong China; 2 Department of Physiology and Pathophysiology, Peking University Health Science Center, Beijing, 100191 China; 3 State Key Laboratory of Ophthalmology, Zhongshan Ophthalmic Center, Sun Yat-Sen University, Guangzhou, 510064 Guangdong China

**Keywords:** Diabetic retinopathy, Retinal neurodegeneration, Retinal ganglion cells, Hyperphosphorylated tau, GSK3β, Synaptic and mitochondrial dysfunction

## Abstract

**Background:**

Although diabetic retinopathy (DR) has long been considered as a microvascular disorder, mounting evidence suggests that diabetic retinal neurodegeneration, in particular synaptic loss and dysfunction of retinal ganglion cells (RGCs) may precede retinal microvascular changes. Key molecules involved in this process remain poorly defined. The microtubule-associated protein tau is a critical mediator of neurotoxicity in Alzheimer’s disease (AD) and other neurodegenerative diseases. However, the effect of tau, if any, in the context of diabetes-induced retinal neurodegeneration has yet to be ascertained. Here, we investigate the changes and putative roles of endogeneous tau in diabetic retinal neurodegeneration.

**Methods:**

To this aim, we combine clinically used electrophysiological techniques, i.e. pattern electroretinogram and visual evoked potential, and molecular analyses in a well characterized high-fat diet (HFD)-induced mouse diabetes model in vivo and primary retinal ganglion cells (RGCs) in vitro.

**Results:**

We demonstrate for the first time that tau hyperphosphorylation via GSK3β activation causes vision deficits and synapse loss of RGCs in HFD-induced DR, which precedes retinal microvasculopathy and RGCs apoptosis. Moreover, intravitreal administration of an siRNA targeting to tau or a specific inhibitor of GSK3β reverses synapse loss and restores visual function of RGCs by attenuating tau hyperphosphorylation within a certain time frame of DR. The cellular mechanisms by which hyperphosphorylated tau induces synapse loss of RGCs upon glucolipotoxicity include i) destabilizing microtubule tracks and impairing microtubule-dependent synaptic targeting of cargoes such as mRNA and mitochondria; ii) disrupting synaptic energy production through mitochondria in a GSK3β-dependent manner.

**Conclusions:**

Our study proposes mild retinal tauopathy as a new pathophysiological model for DR and tau as a novel therapeutic target to counter diabetic RGCs neurodegeneration occurring before retinal vasculature abnormalities.

**Electronic supplementary material:**

The online version of this article (10.1186/s13024-018-0295-z) contains supplementary material, which is available to authorized users.

## Background

Although diabetic retinopathy (DR) has long been regarded as a microvascular disorder, it has now become evident that inner retinal neurodegeneration, including electroretinogram (ERG) abnormalities, ganglion cells loss, reactive gliosis, and inner retinal thinning [1] also occurs in people with DR. Remarkably, mounting evidence shows that neuroretinal alterations are present even in the absence of any visible microvascular signs of DR [2, 3]. These observations support the concept that DR may be a neurodegenerative eye disease occurring in the early phase of DR [4].

Studies show that retinal ganglion cells (RGCs) exhibit the greatest level of dysfunction within the components of ERG in rat model of DR [5]. As the sole neurons that convey visual information from the retina to the brain, the structural and functional integrity of RGCs is crucial for preserving visual function. A significant increased apoptosis and loss of RGCs were reported in diabetes [6, 7]. Intriguingly, suffering of RGCs from diabetes has also been demonstrated by the decrease in synaptic proteins in retinal nerve terminals [8] and by reduced efficiency of both retrograde and anterograde RGC axonal transport [9], suggesting that vision deficit as revealed by ERG may be due to impaired synaptic function starting well before the appearance of apoptosis [10]. Nevertheless, key molecules involved in synapse degeneration in DR are still poorly defined.

The microtubule-associated protein tau is a critical mediator of neurotoxicity in Alzheimer’s disease (AD) and other neurodegenerative diseases, with functions in assembly and stabilization of microtubules, axonal transport, and neurite outgrowth in a phosphorylation-dependent manner [11]. This function of tau can be compromised by abnormal hyperphosphorylation [12]. A growing body of studies reveals that synapse loss is strongly correlated with the presence of increased phosphorylated tau in AD brain [13, 14]. Hyperphosphorylated tau has also recently been detected in the RGC layer and associated with neuroretinal dysfunction in transgenic mice carrying the P301S mutant human tau [15], proposing a correlation between tau hyperphosphorylation and RGCs degeneration. It is increasingly recognized that diabetes shares a number of pathological features with AD. In support of this, animal models for diabetes show AD-like changes in brain, including exacerbated tau phosphorylation and synapse degeneration [16, 17]. However, whether endogenous tau contributes to diabetic retinal neurodegeneration remains currently unknown.

The rodent models used to study DR include spontaneous models such as the *db*/*db* mice, and streptozotocin (STZ)- or diet-induced diabetes models. A disadvantage of STZ administration in examining retinal neurodegeneration is its off-target neurotoxic effects [18, 19]. Given that leptin signaling has an important trophic effect in the retina, a leptin-receptor mutation in *db*/*db* mice might conceal a potential early neural contribution to visual dysfunction seen in these mice. In this study, we investigated the changes and putative roles of tau in the electrophysiological and synaptic dysfunction of RGCs in a high-fat diet (HFD)-induced diabetes mouse model which exhibits the pathophysiological features of the slow-onset retinopathy of type 2 diabetes [2, 20–22]. We demonstrate for the first time that abnormal tau hyperphosphorylation via GSK3β activation causes visual deficits of RGCs through the disruption of synaptic and mitochondrial functions in the context of HFD-induced diabetic RGCs neurodegeneration.

## Methods

### Animals

Male C57BL/6 mice used in this study were purchased from Guangdong Medical Laboratory Animal Center (Guangzhou, China). Starting at 5 weeks of age, mice were fed either a HFD (45% fat calories, 20% protein calories, and 35% carbohydrate calories; Mediscience Ltd., China) as previously described [23] or regular standard laboratory chow (RD) as controls (10% fat calories, 20% protein calories, and 35% carbohydrate calories; Mediscience Ltd) for up to 24 weeks. Their body weight and food intake were monitored throughout.

### Metabolic parameters

Glucose and insulin tolerance tests were performed after an 8 h fast by an oral administration of _D_-glucose (1 g/kg in 0.9% NaCl) (OGTT) or by an intraperitoneal injection of insulin (0.75 unit/kg, Gibco, Life Technologies, Grand Island, NY, USA) (IPITT), respectively. Blood glucose levels were detected from the tail vein immediately before (0 min) and at 30, 60, 90, and 120 min after glucose or insulin administration. Levels of circulating serum triglyceride (TG) and free fatty acids (FFA) were determined with a commercial kit (Wako Chemicals, Osaka, Japan) according to the manufacturer’s instruction. Levels of fasting serum insulin were assayed by an Insulin (Mouse) Ultrasensitive ELISA kit (ALPCO Diagnostics, CA, USA).

### Pattern ERG (PERG) and visual evoked potential (VEP)

Mice were dark-adapted, their pupils dilated, and anesthetized by intraperitoneal injection with ketamine (75 mg/kg) and xylazine (10 mg/kg). PERG and VEP were performed using a RETI-Port/Scan 21 recorder (Roland Consult, Wiesbaden, Germany) according to the manufacturer’s instructions. For PERG, pattern stimuli consisted of horizontal, square grating bars of constant luminance, contrast, and spatial and temporal frequencies. The standard grating contrast was 90%, with a 1 Hz reversal rate, and spatial frequency of 0.05 cycle/deg. For VEP, responses were elicited by 300 consecutive flash stimuli with an intensity of 500 cd·sec/m2 for 2 min to one eye.

### Vascular permeability assays

Fundus fluorescein angiography (FFA). Under anesthesia, mice were intravenously administered with sodium fluorescein (5 mg/kg of 10% *w*/*v*, Akorn, Lake Forest, IL). Fundus angiograms were obtained 2 min later at 490 nm with a Micron III camera (Phoenix, Pleasanton, CA, USA).

Retinal Evans Blue (EB) angiography. Briefly, mice were anesthetized; the descending aorta was clamped and then perfused via the left ventricle with 1 ml warm PBS containing EB dye (25 mg/kg of 1% solution, Sigma-Aldrich, CA, USA). The eyes were enucleated and placed in 4% paraformaldehyde for 2 h. Retinae were then dissected, flat-mounted in fluorescent mounting medium, and imaged with at 620 nm with a Leica DMI4000B inverted fluorescence microscope (Leica Microsystems, Wetzlar, Germany).

### Immunohistochemistry

For retina immunohistochemistry study, eyes were rapidly dissected and fixed in 4% paraformaldehyde at 4 °C for 48 h, embedded in paraffin for the generation of thin retinal cross sections (4 μm) using a microtome (Leica Biosystems). For primary RGCs immunohistochemistry study, cells cultured on glass coverslips were fixed with 4% paraformaldehyde and permeabilized in 0.1% TritonX-100. After blocking with 5% normal goat serum, the retinal sections or cells were incubated overnight at 4 °C with primary antibodies (Table 1). Immunoreactivity was visualized using secondary antibodies conjugated with Alexa-Fluor-chromes (Invitrogen™, Life Technology, MA, USA), including Alexa-Fluor-488 goat anti-rabbit, − 594 goat anti-mouse, and − 405 goat anti-chicken IgG (1:100). Nuclei were counterstained with 4′,6-diamidino-2-phenylindole (DAPI; Invitrogen). Images were obtained with a confocal microscope (LSM 510 Meta; Carl Zeiss, Oberkochen, Germany). For quantitative immunohistochemistry, a line moving vertically from GCL to OPL across the images measured the gray values after uniform background subtraction using Plot Profile function in Image J. Results obtained from three independent observed areas of each retinal section were averaged and plotted in curvilineal diagrams using Origin 8.0 software. The immunostaining intensity was quantified by measuring the area under the curve for GCL and IPL with Origin 8.0. For each eye, data from three independent curvilineal diagrams were averaged, and the mean of five eyes was used as the representative value for each group.

**Table 1:** Primary antibody information

Antibody	Host	Application	Source	Cat. No
β-tubulin	Rabbit	WB(1:1000)	CST^a^	#2146
Ac-Tubulin	Rabbit	WB(1:1000)/IHC(1:100)	CST	#3971
Akt(pan)	Rabbit	WB(1:1000)	CST	#4691
pS473-Akt	Rabbit	WB(1:1000)	CST	#4060
Cleaved caspase-3	Rabbit	WB(1:1000)	CST	#9664
Complex I	Rabbit	WB(1:1000)/IHC(1:100)	Abclonal	A7862
Complex IV	Rabbit	WB(1:1000)	Abclonal	A6564
GAPDH	Mouse	WB(1:3000)	Abclonal	AC033
GSK3β	Rabbit	WB(1:1000)	CST	#9315
pS9-GSK3β	Rabbit	WB(1:1000)	CST	#9322
IRS1	Rabbit	WB(1:1000)	CST	#2382
MAP2	Chicken	IHC(1:10000)	Abcam	ab75713
Synaptophysin	Rabbit	WB(1:5000)/IHC(1:100)	Abcam	ab32127
Tau-1	Mouse	WB(1:5000)/IHC(1:100)	EMB Millipore	MAB3420
Tau-5	Mouse/ Rabbit	WB(1:5000)/IHC(1:100)	Abcam/ Beyotime	Ab80579/ AF1249
pT205-Tau	Rabbit	WB(1:1000)/IHC(1:100)	Abclonal	AP0168
pT231-Tau	Rabbit/ Mouse	WB(1:1000)/IHC(1:100)	Abclonal/ Abcam	AP0401/ ab89748
pS396-Tau	Rabbit	WB(1:1000)/IHC(1:100)	Abclonal	AP0163
pS404-Tau	Rabbit	WB(1:1000)/IHC(1:100)	Abcam	ab92676
Thy1	Mouse	IHC(1:100)	Abcam	ab225
TUJ1	Rabbit	IHC(1:100)	Abcam	Ab18207
Tyr-tubulin	Mouse	IHC(1:100)	Sigma-Aldrich	T9028

^a^*CST* Cell Signaling Technology

### TUENL assay

TUNEL assay was performed according to the manufacturer’s instructions (Roche, USA). Nuclei were counterstained with DAPI and the sections were mounted using Dako glycergel mounting medium. Representative images were acquired with a Carl Zeiss LSM 510 Meta confocal microscope. For each retinal section, the number of TUNEL positive cells in the GCL was counted. For each eye, results obtained from four independent sections were averaged, and the mean of six eyes was used as the representative value for each group.

### Golgi staining of RGCs axons

Optic nerves were rapidly dissected from mice and processed for Golgi staining using a FD Rapid GolgiStain™ Kit (FD NeuroTechnologies, MD, USA) according to the manufacturer’s instructions. After staining, optic nerves were embedded in optimal cutting temperature compound (Tissue-Tek, Leica, Nussloch, Germany) for the generation of longitudinal cryosections (50 μm) that were collected onto gelatin-coated slides. Golgi staining of RGCs axons at the proximal portions of optic nerves was detected under bright field and polarized light.

### Western blot analysis

Western blotting was performed as previously described [24] using primary antibodies indicated in Table 1. HRP-conjugated secondary antibodies (1:2000) were from Cell Signalling Technology. Immunoreactive bands were revealed by enhanced chemiluminescence (SuperSignal™ West Pico Chemiluminescent Substrate kits, Thermo Scientific) and visualized by the KODAK Image Station 4000MM PRO. Band intensities were quantified by scanning densitometry (Gel-Doc2000, Bio-Rad) and analyzed with Quantity One™ (Bio-Rad).

### Short interfering RNA (siRNA)

To knock-down of tau in retinae, the following siRNA sequences (sense strands) against tau (si-Tau) used in a previous study [25] were purchased from Dharmacon (ON-TARGET plus Smart-pool, Thermo Scientific): 1) 5’-GCAUGUGACUCAAGCUCGA-3′; 2) 5’-AGUUAGGGACGAUGCGGUA-3′; 3) 5’-GAUAGAGUCCAGUCGAAGA-3′; 4) 5’-GGACAGGAAAUGACGAGAA-3′. These sequences are highly conserved between mouse and rat. In addition, si-Tau sequence 4) conjugated with Cy5 was adopted for knock-down of tau in primarily cultured RGCs. A scramble siRNA sequence conjugated with Cy5 was used as a scramble control (si-sc). To knock-down of GSK3β in retinae, the siRNA sequence (sense strand) against GSK3β (si- GSK3β) was used: 5’-CCACTCAAGAACTGTCAAGTA-3′. The siRNA was purchased from Genemall (Shenzhen, China). A scramble siRNA sequence was used as a scramble control (si-sc): 5’-UUCUCCGAACGUGUCACGUTT-3′.

### Intravitreal injection

Intravitreal injections were performed as previously described [15, 25]. Briefly, mice were anesthetized by intraperitoneal injections of 2 g/kg urethane. Pupils were dilated with 1% atropine sulfate. Intravitreal injections were performed using a Hamilton syringe fitted with a 30-gauge glass microneedle under a dissecting microscope. One microliter of the solution was slowly injected into the vitreous chamber of the eye. For selected tau or GSK3β depletion, si-Tau or si-GSK3β (2 μg/μl) was injected intravitreally into the right eye and si-sc was injected into the left eye. For GSK3β inhibition, a GSK3β specific inhibitor TWS119 (Selleck, Trenton, NJ, USA; 10 nM final concentration), or vehicle (0.5% DMSO) was dissolved in 0.9% NaCl and injected intravitreally into the right eye or left eye, respectively.

### Isolation, culture and treatment of primary RGCs

Neonatal Sprague-Dawley rats (1–3 days old) were purchased from Guangdong Medical Laboratory Animal Center. Primary neonatal rat RGCs were isolated using a Thy-1 antibody-panning method adapted from a previous study [26]. Briefly, the isolated retinae from neonatal rats were digested with 4.5 units/mL of papain solution (Worthington, Lakewood, NJ, USA) to dissociate the cells. Cell suspensions were purified by sequential immunopanning with a rabbit anti-macrophage and anti-Thy1 antibody (Abcam), respectively. Cells were then dissociated by trypsin treatment (1250 U/mL; Sigma-Aldrich) from the Thy-1 antibody-coated dish. Dissociated cells were seeded on glass coverslips precoated with poly-_D_-lysine (Sigma-Aldrich) in serum-free Neurobasal-A medium (Gibco) supplemented with 1% B27 (Gibco), 1% glutamine (Invitrogen), 5 mM of _D_-glucose, and 50 U/mL of penicillin-streptomycin. One-half of the culture medium was replaced every two days. Cultured RGCs at day 5 were identified by analyzing the expression of RGC-characteristic marker Thy1, as well as neuronal markers TUJ1 and Map2 by immunofluorescence. Alternatively, after 5 or 6 days in culture, RGCs were treated with conditioned medium containing 10 mM of _D_-gulcose and 200 μM of palmitate (Sigma-Aldrich, MO, USA) pre-conjugated to BSA (Sigma–Aldrich) or control medium containing 5 mM of _D_-glucose and 5 mM mannitol, in the absence or presence of TWS119 (10 nM), for 24 h. For tau knock-down experiments, cultured RGCs at day 5 were transfected by si-Tau or si-sc with a Micropoly-transfecter™ cell reagent (Biosky, China) according to the manufacturer’s instructions. Twenty-four hours after transfection, cells were subjected to conditioned medium for another 24 h.

### Isolation of synaptosomes from primary RGCs

Synaptosomes were isolated from primary RGCs using a protocol adapted from a previously study [27]. Cell pellets in 1 ml of fresh sucrose buffer were vortexed and centrifuged at 200 *g* for 10 min at 4 °C to pellet nuclear elements. The resulting supernatant was centrifuged at 800 *g* for 12 min at 4 °C to isolate cytoskeletal components. The synaptosome fraction was harvested by centrifugation the resulting supernatant at 25,000 *g* for 14 min at 4 °C. Synaptosome pellets were washed twice in 1 ml of ice-cold sucrose buffer to remove the contamination, and reharvested by centrifugating at 25,000 *g* for 14 min at 4 °C. Synaptosome fractions were confirmed by immunoblot analysis.

### Microtubule sedimentation assay

Microtubule sedimentation assay was performed as described previously [28]. Briefly, cultured RGCs were lysed by sonication in 0.1 M PIPES (pH 6.6), 1 mM EGTA, 1 mM MgSO4, 1 mM β-mercaptoethanol, 0.2% NP-40, in the presence of protease and phosphatase inhibitors. Protein lysate at a final concentration of 1 mg/ml was mixed with 1 mM GTP and 10 μM taxol and incubated at 37 °C for 30 min. After centrifugation, supernatants and pellets were harvested and analyzed by Western blot analysis.

### Mitochondrial mobility assay

Mitochondrail axonal trafficking in primarily cultured RGCs was determined as previously described [29]. Mitochondria were labeled by infecting cultured RGCs at day 5 with an adenovirus vector carrying the complex IV gene (Ad-GFP-Mito), followed by the respective treatment. RGCs were imaged 48 h after infection. A process 2–3 times longer than other processes was considered as an axon. Mitochondria were identified as particles with strong GFP labeling within axons. A series of time lapse photography was captured every 5 s with an OLYMPUS FV1000 confocal microscope for a total of 2.5 min and saved as avi files. Kymographic images showing the overall trafficking of mitochondria were generated by using ImageJ software with a Mulitple Kymograph plug-in. Mitochondrial movement were analyzed from the kymographs. Each series of images was taken for three randomly selected GFP-mito-labeled cells per culture and three independent cultures per treatment.

### Mitochondrial complex activity assay

Mitochondria were isolated from 5 × 10^6^ primary RGCs using the Cell Mitochondria Isolation Kit (Beyotime Co., China). The activities of the mitochondrial complex I or IV were determined by the Micro Mitochondrial Respiratory Chain Complex I or IVActivity Assay Kit (Solarbio, China) according to the manufacturer’s instructions. Briefly, mitochondrial homogenates were added into the respective reaction buffer. The reaction mixture was transferred to a prewarmed (30 °C) quartz cuvette and immediately put into a spectrophotometer. The absorbance of reaction mixture was measured at 340 nm for Complex I, or 550 nm for Complex IV, respectively. Mitochondrial complex activity was expressed as nmol/min/mg protein.

### Quantitative real-time polymerase chain reaction (Q-PCR)

The RNA from whole cell pellets or synaptosome pellets was extracted using the RNeasy Mini Kit (Qiagen, Germany) according to the manufacturer’s instructions. Reverse transcription was performed with Random hexamers and SuperScript-III (Invitrogen). Quantitative real-time PCR was carried out with the Applied Biosystems 7300 real-time systems using real-time PCR Master Mix (SYBR Green). The primer sequences were as follows: synaptophysin sense, 5’-AGACATGGACGTGGTGAATCA-3′; antisense, 5’-ACTCTCCGTCTTGTTGGCAC-3′; GAPDH sense, 5′- AGCAGTCCCGTACACTGGCAAAC-3′; antisense, 5′- TCTGTGGTGATGTAAATGTCCTCT-3′.. Each experiment was performed in triplicate in three independent experiments.

### Statistical analysis

All data were expressed as mean ± SEM. Statistical significance was analyzed by one-way ANOVA followed by a Student’s *t* test. Data were considered significant when *P* < 0.05.

## Results

### Electrophysiological deficit of RGCs precedes retinal microvascular changes in HFD-induced diabetes

**Fig. 1:**
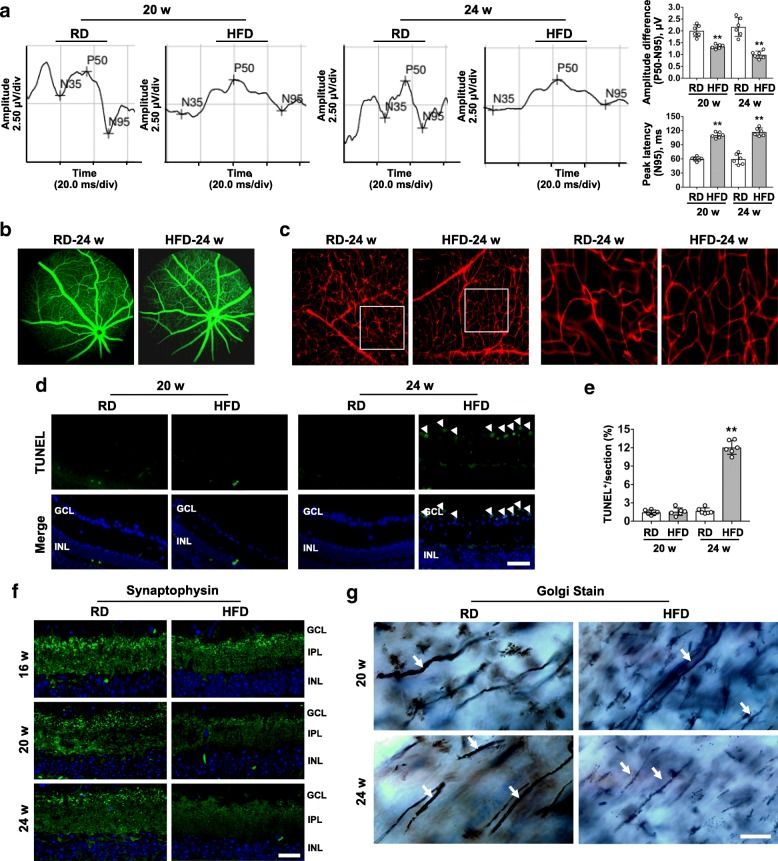
Decreased RGCs activity in the absence of retinal microvasculopathy is associated with synaptic and axonal impairment occurring before RGCs apoptosis in HFD-induced diabetes. (**a**) Representative pattern electroretinography (PERG) waveforms of eyes in mice fed with regular chow (RD) or HFD for 20 and 24 weeks. Difference between peak-to-peak amplitude of P50 and N95 components (P50-N95) and the N95 peak latency were quantified. (**b**) Representative images of fundus fluorescein angiography. (**c**) Illustrative examples of retinal Evans Blue angiography. Right panels are high-power magnification of the areas indicated by the boxes. (**d**) Representative images of retinal immunostaining for apoptotic (TUNEL positive, green; indicated by arrow) cells. Nuclei were labeled with DAPI (blue). Scale bar, 100 μm. (**e**) Apoptotic RGCs were quantified and expressed as the percentage of TUNEL-positive cells to DAPI-positive cells in GCL. For each retinal section, the number of TUNEL positive cells in the GCL was counted. For each eye, results obtained from four independent sections were averaged. (**f**) Retinal immunofluoresence staining for synaptophysin (green, synaptophysin; blue, DAPI; scale bar, 100 μm). (**g**) Representative images for Golgi staining of RGCs axons at the proximal portions of optic nerves (black, indicated by arrow; scale bar, 10 μm) in longitudinal cryosections of optic nerves. Data are means ± SEM. *n* = 6 animals per group. ^**^P < 0.01 vs age-match RD controls. GCL, ganglion cell layer; IPL, inner plexiform layer; INL, inner nuclear layer

Mice fed a HFD for 16 weeks exhibited obesity in association with early type 2 diabetic conditions, which persisted through 24 weeks of diet feeding (Additional file 1: Figure. S1a-g). To determine whether HFD-induced diabetes affects RGCs function, we used PERG to measure the response of the retina to patterned light stimuli that specifically elicit RGCs activity [30]. Difference between peak-to-peak amplitude (P50-N95) was significantly reduced, while the N95 peak time (latency) was significantly increased in mice fed with HFD for 20 or 24 week, as compared to age-matched controls (Fig. 1a), suggesting that HFD for 20 weeks is sufficient to trigger significant RGCs dysfunction. Further, to investigate whether microvascular lesions are associated with HFD-induced visual deficits, we analyzed the retinal vascular permeability by fundus fluorescence angiography (FFA) and EB dye leakage assay. Intriguingly, even HFD for 24 weeks failed to induce increased permeability of retinal vessels (Fig. 1b,c), which was found to occur after HFD for 12 months (Additional file 1: Figure. S1j), in consistent with a previous study [2]. Thus, our data suggest that electrophysiological abnormalities of RGCs precede the development of visible retinal microvascular lesions in a diabetes model induced by a HFD feeding.

### HFD-induced synapse loss and axonal impairment occur before apoptosis in RGCs

We first investigated whether HFD-induced RGCs dysfunction is attributed to apoptotic death. There was no evidence of RGCs apoptosis in the retina until 24 weeks, when mice fed with HFD showed a significant increase in TUNEL-positive nuclei (Fig. 1d,e) and caspase-3 activation (Additional file 1: Figure. S1 h) in the ganglion cell layer (GCL) which is the location of RGCs soma. This finding indicates that compromised activity of RGCs induced by HFD feeding for as early as 20 weeks is not driven by RGCs apoptosis. We then analyze the expression of synaptophysin, an abundant vesicular protein that modulates presynaptic neurotransmitter and regulates vesicle recycling. Retinal sections from HFD mice had considerably less synaptophysin-immunoreactive puncta in the inner plexiform layer (IPL), where RGCs dendrites are located, after both 20 and 24 weeks of feeding, with comparison to age-matched controls (Fig. 1f). Immunoblotting for whole retina lysates also revealed significantly reduced synaptophysin in mice fed with HFD for 20 and 24 weeks (Additional file 1: Figure S1i). Moreover, Golgi staining of proximal optic nerve organized by axon bundles of RGCs showed axonal thinning in mice fed with HFD for 20 and 24 weeks, respectively (Fig. 1g). These results suggest that HFD-induced synapse loss and axonal impairment may contribute to reduced activity of RGCs in early phase of DR, when apoptosis of RGCs has not yet been developed.

### HFD promotes hyperphosphorylation of tau in retinae

**Fig. 2:**
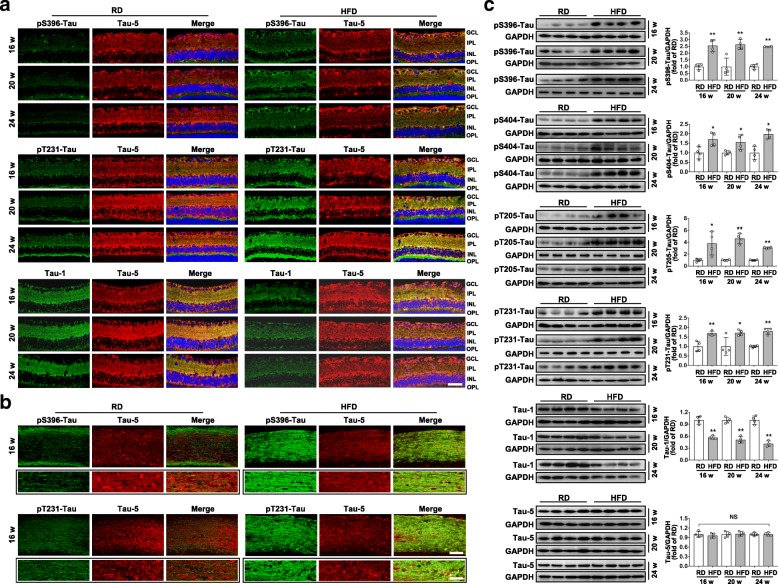
HFD promotes hyperphosphorylation of tau in neural retina and optic nerves. (**a**) Representative images of retinal double immunostaining for phospho-tau (pS396- or pT231-Tau; green) with total-tau (Tau-5; red), and non-phospho-tau (Tau-1; green) with Tau-5 (red) from mice fed with RD or HFD for 16, 20 and 24 weeks, respectively. Nuclei were counterstained with DAPI. Scale bar, 100 μm. (**b**) Representative images of double immunostaining for phospho-tau (pS396- or pT231-Tau; green) with total-tau (Tau-5; red) of RGCs axons at the proximal portions of optic nerves in longitudinal sections of optic nerves from mice fed with RD or HFD for 16 weeks. Scale bar, 20 μm. Higher magnifications (scale bar, 5 μm) are shown in the boxed lower panels, respectively. (**c**) Western blot analyses of pS396-Tau, pS404-Tau, pT231-Tau, pT205-Tau, Tau-1 and Tau-5 in total retina lysate. Intensities were quantified and normalized against the level of GAPDH and expressed as fold changes of protein abundance in the retina from HFD groups relative to their age-matched controls. Data are means ± SEM. n = 6 (a, b) or *n* = 4 mice (b) per group. ^*^P < 0.05 and ^**^P < 0.01 vs age-match RD controls. NS, no significant difference. OPL, outer plexiform layer

We then investigated whether HFD-induced RGCs’ synaptic degeneration and visual dysfunction are attributed to abnormal tau hyperphosphorylation. Relative to age-matched controls, mice fed with HFD for 16 weeks showed a ubiquitously increased hyperphosphorylation pattern of tau at residues of serine 396 (S396) and 404 (S404), threonine 205 (T205) and 231 (T231), which define AD’s type of phospho-tau [31], primarily in the GCL and IPL of retinae (Fig. 2a; Additional file 1: Figure. S2a), and in the proximal optic nerve (Fig. 2b; Additional file 1: Figure. S2b). The increase in tau hyperphosphorylation persisted through HFD feeding for 24 weeks, which was accompanied by a decrease in non-phosphorylated tau at residues of S195/S198/S199/S202 as detected by Tau-1 (Fig. 2a). Immunoblotting of retinal homogenates confirmed a significantly increased level of phosphorylated tau and reduced level of non-phosphorylated tau in mice fed with HFD as early as for 16 weeks (Fig. 2c). Interestingly, no significant changes of total tau (Tau-5) were detected between age-matched groups.

### Knock-down of tau rescues RGCs dysfunction and synapse loss in mice fed a HFD

**Fig. 3:**
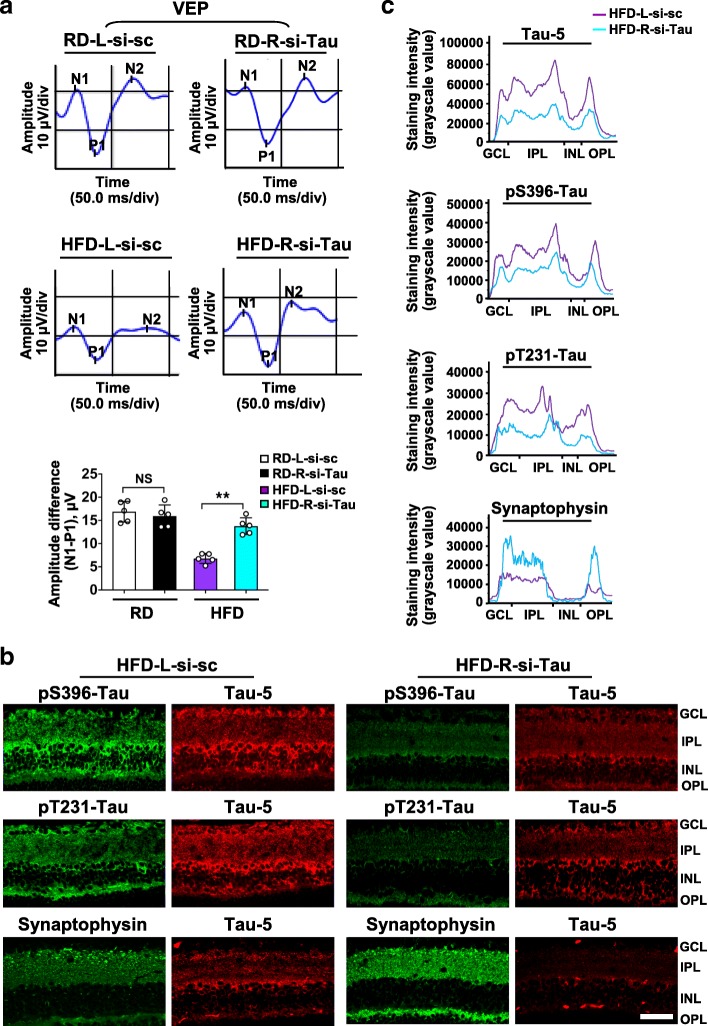
Intravitreal delivery of si-Tau restores HFD-induced RGCs dysfunction and synapse loss**.** si-Tau was injected in the vitreous of the right (R) eye of mice at 20 weeks after RD (RD-R-si-Tau) or HFD (HFD-R-si-Tau), while a scramble si-sc was injected in the contralateral (left; L) eye as a control (RD-L-si-sc; HFD-L-si-sc). (**a**) Representative waveforms of visual evoked potential (VEP). The differences in peak amplitude (N1-P1) were quantified. (**b**) Representative images of double immunostaining for phospho-tau (pS396- or pT231-Tau; green) with total-tau (Tau-5; red), and for synaptophysin (green) with Tau 5 (red). Scale bar, 100 μm. (**c**) Representative curvilineal profile of protein immunostaining intensity from GCL to OPL across retinal depth of the images shown in B. Data are means ± SEM. *n* = 5 eyes per group. For each eye, data from three independent curvilineal diagrams were averaged, and the mean of five eyes was used as the representative value for each group. ^**^P < 0.01 vs contralateral eye injected with si-sc. NS, no significant difference

To elucidate whether abnormal hyperphosphorylation of tau per se mediates synaptic loss and reduced activity of RGCs in HFD-fed mice, an siRNA-based strategy was chosen based on the capacity that 1) siRNA delivered by intravitreal injection is rapidly taken up by RGCs [32]; 2) selective suppression of tau reduces the total amount of retinal tau protein available for phosphorylation. The knock-down efficiency with a targeted si-Tau [25] was confirmed one week after a single intravitreal injection (Additional file 1: Figure. S3a). We next investigated the effect of siRNA-mediated tau repression on visual function in mice fed with HFD for 20 weeks, a time when significantly impaired RGCs activity occurred. For this purpose, si-Tau was injected in the vitreous of mice at 20 weeks after HFD, while scramble si-sc was injected in the contralateral eye as a control. One week later, the physiological function of both RGC somata and axons was evaluated by VEP [33]. Strikingly, eyes treated with si-Tau in HFD-fed mice showed significantly improved visual responses, with a ~ 2-fold increase in peak amplitude differences (N1-P1), compared with contralateral eyes treated with si-sc. In contrast, intraocular injection of si-Tau in eyes of age-matched RD controls did not produce detrimental effects on VEP responses with comparison to eyes injected with si-sc (Fig. 3a). Further, we harvested the retina from mice underwent VEP for immunostaining. Knock-down of tau in eyes of age-matched RD controls did not show destructive effects in comparison with si-sc control (Additional file 1: Figure. S3b and c), suggesting that tau downregulation does not cause disease phenotype under physiological conditions as described [25]. However, in a HFD-induced diabetes setting, eyes treated with si-Tau displayed significantly reduced immunoreactivity for total and phosphorylated tau, accompanied by distinctly increased expression of synaptophysin, as compared with si-sc control. (Fig. 3b,c; Additional file 1: Figure. S4a-c). Collectively, these data demonstrate that phospho-tau is requisite for HFD-induced visual deficits and synapse loss.

### Attenuated tau hyperphosphorylation by GSK3β inhibition protects RGCs from HFD-induced vision and synapse loss

**Fig. 4:**
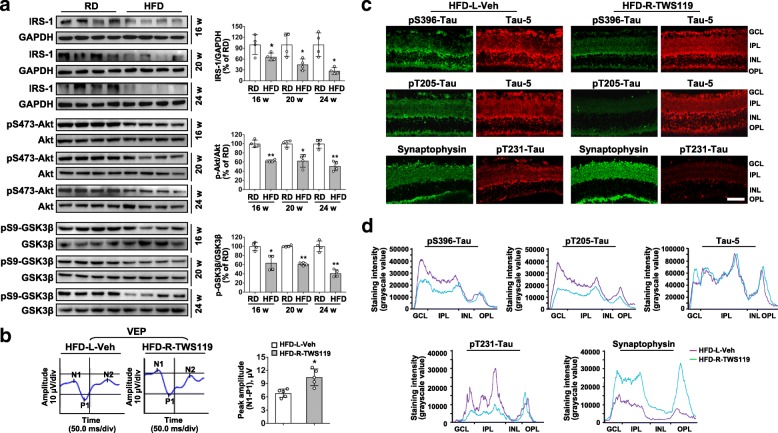
Reduced tau phosphorylation by intravitreal injection of a GSK3β inhibitor protects RGCs from HFD-induced vision and synapse loss. (**a**) Western blotting for IRS-1, phosphorylated-Akt (S473), total Akt, phosphorylated-GSK3β (Ser9), total GSK3β in total retina lysate. Intensities were quantified and normalized against the level of GAPDH or total proteins (Akt or GSK3β) and expressed as percentage of protein abundance in the retina from HFD groups relative to their age-matched controls. Data are means ± SEM. n = 4 mice per group. ^*^P < 0.05 and ^**^P < 0.01 vs age-match RD controls. (**b**) A GSK3β specific inhibitor TWS119 or vehicle was injected intravitreally into the right eye (HFD-R-TWS119) or left eye (HFD-L-Veh), respectively, in mice fed with HFD for 20 weeks. Representative VEP waveforms and quantification of differences in peak amplitude (N1-P1) are shown. (**c**) Representative double immunostaining for phopho-tau (pS396- or pT205-Tau; green) with Tau-5 (Red), and for synaptophysin (green) with pT231-Tau (red). Scale bar, 100 μm. (**d**) Representative curvilineal profile of protein immunostaining intensity from GCL to OPL across the image shown in **c**. Data are means ± SEM. n = 5 eyes (b-d) per group. ^*^P < 0.05 vs contralateral eye injected with vehicle

We next determined whether tau hyperphosphorylation in degenerated RGCs is resulted from an activation of glycogen synthase kinase 3β (GSK3β), a predominant tau kinase, and found a decrease in the inactivating phosphorylation (pSer9)-GSK3β in retinae from mice fed a HFD. Moreover, there was a significant downregulation of phospho-Akt (Ser473), a key mediator in insulin signaling, which phosphorylates GSK3β at Ser9 and keeps it inactive, and IRS-1, a target of free fatty acids in insulin resistance, in retinae from mice with HFD (Fig. 4a), suggesting that abnormal hyperphosphorylation of tau in retinae may be attributed to HFD-induced dysregulation of IRS1/Akt/GSK3β signaling. To further explore the role of GSK3β in HFD-induced visual impairment, we injected a GSK3β inhibitor, TWS119 (10 nM), or vehicle in the vitreous of mice fed with HFD for 20 weeks and analyzed the RGCs function 24 h after injection by VEP. Eyes treated with TWS119 showed a significant improved visual response, compared to vehicle treated contralateral eyes (Fig. 4b). Alternatively, an siRNA targeted to GSK3β (si-GSK3β) was intravitreally injected in mice at 20 weeks after HFD, while scramble si-sc was injected in the contralateral eye as a control. Knock-down of GSK3β also considerably improved VEP responses a week after injection, with comparison to control eyes (Additional file 1: Figure S6a). We further found that inhibition of GSK3β in eyes from HFD-mice did not affect the expression of total tau, but considerably attenuated tau hyperphosphorylation and restored synaptophysin expression, as compared to vehicle or si-sc treated contralateral eyes, respectively (Fig. 4c,d; Additional file 1: Figure. S5a-c; Figure. S6b-e). Interestingly, selected inhibition of GSK3β in eyes of age-matched RD controls by TWS119 did not show detrimental effects with comparison to vehicle treated contralateral eyes (Additional file 1: Figure. S7a-c). Taken together, these results indicate that activation of GSK3β by impaired IRS1/Akt signaling is required for tau hyperphosphorylation in a HFD-induced DR model.

### Glucolipotoxicity reduces tau microtubule binding and destabilizes microtubules through activation of GSK3β in primary RGCs

**Fig. 5:**
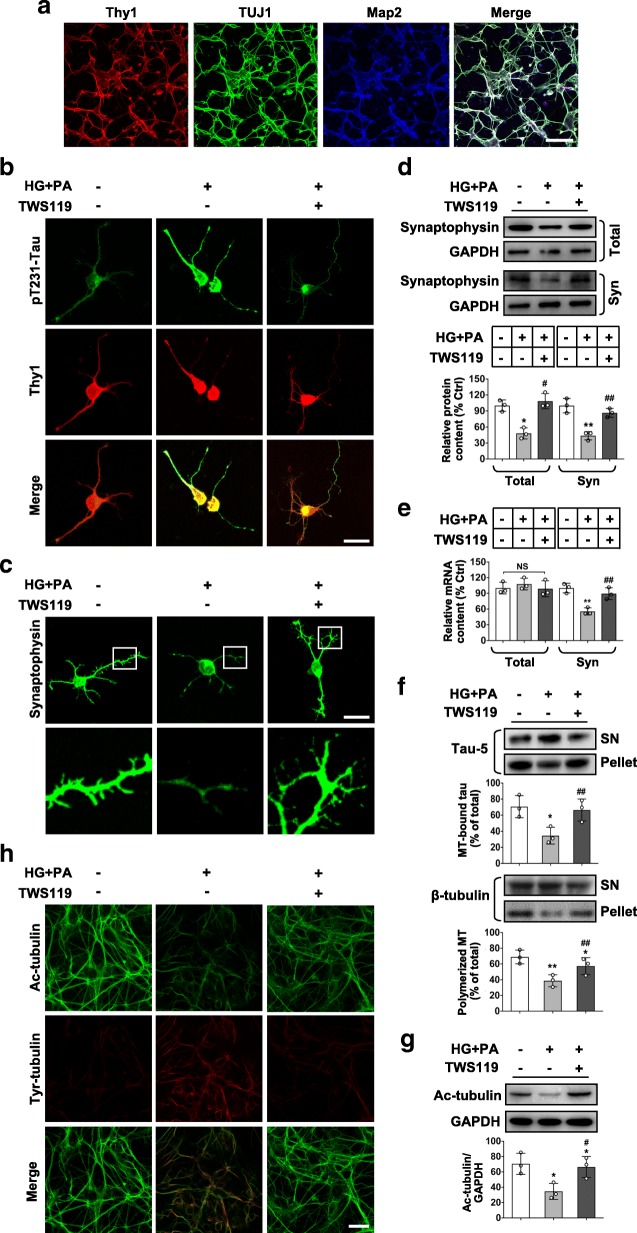
Reduced tau microtubule binding and microtubule stability are associated with synapse loss in primary RGCs upon glucolipotoxicity in a GSK3β-dependent manner. (**a**) Representative images of triple immunostaining for RGC-characteristic marker Thy1 (red), neuronal markers TUJ1 (green) and Map2 (blue). Scale bar, 100 μm. Primary RGCs were then exposed to conditioned medium (HG + PA) for 24 h, in the absence or presence of TWS119. (**b**) Representative images of subcellular expression of pT231-Tau (green) and Thy1 (red) by double immunofluorescence. Scale bar, 20 μm. (**c**) Representative synaptophysin (green; scale bar, 20 μm) immunostaining in RGCs. Areas boxed in are shown at higher magnification in the lower panels. (**d**) Western blotting for synaptophysin from whole cell lysates (Total) or synaptosome fractions (Syn). Intensities were quantified and normalized against the level of GAPDH and expressed as percentages of protein abundance under stimulation relative to control. (**e**) mRNAs of synaptophysin in total lysates or synaptosomes were quantified by Q-PCR. (**f**) Microtubule sedimentation assay. Western blotting for Tau 5 and β-tubulin in the supernatant (SN) and the microtubule pellet (pellet). Relative intensities of each protein in its respective fraction were quantified and normalized against the sum of the intensity value of that protein (total, including both supernatant and pellet fractions). MT, microtubule. (**g**) Western blotting for Ac-tubulin in whole cell lysates. Intensities were normalized against the level of GAPDH. (**h**) Representative images of double immunofluorescence for Ac-tubulin (green) and Tyr-tubulin (red). Scale bar, 40 μm. Data are means ± SEM of three independent experiments. ^*^P < 0.05 and ^**^P < 0.01 vs control; ^#^*P* < 0.05 and ^##^*P* < 0.01 vs HG + PA

We further illuminated the mechanisms by which tau drives RGCs synaptic degeneration in vitro. Primary neonatal rat RGCs were validated by the expression of the RGC-characteristic marker Thy1, as well as neuronal markers TUJ1 and Map2 (Fig. 5a). Given that the HFD-induced diabetes mice exhibited increased blood levels of free fatty acids and slight hyperglycemia, chronic exposure of RGCs to conditioned medium containing 10 mM of _D_-glucose and 200 μM of BSA-conjugated palmitate was used to produce a model of HFD-induced glucolipotoxicity in vitro. Stimulation of RGCs with conditioned medium for 24 h led to significant activation of GSK3β, hyperphosphorylation of tau, and loss of synaptophysin (Fig. 5b,c; Additional file 1: Figure. S8a-c). Intriguingly, whereas there was a considerable decrease in total protein levels of synaptophysin in RGCs upon glucolipotoxicity (Fig. 5d), the total mRNA content for synaptophysin was not significantly different to controls (Fig. 5e). In contrast, synaptosomes isolated from RGCs under glucolipotoxicity showed significantly lower mRNA and protein levels of synaptophysin than controls (Fig. 5d,e).

We then ask whether the unchanged total mRNA levels but reduced mRNA and protein contents in synaptosome upon glucolipotoxicity may be due to a failure in mRNA and organelles transport from the soma to neurite, thus a decreased local protein synthesis within synapses [34]. To test this possibility, we detected the effect of glucolipotoxicity on tau microtubule binding which is associated with the stability of microtubules essential for axonal/dendritic transport. We found that glucolipotoxicity led to a significant reduction in the amount of tau bound to microtubules and settled in the microtubule pellet, accompanied by decreased levels of polymerized microtubules (Fig. 5f). Accordingly, RGCs under glucolipotoxic stress showed a markedly reduced expression of Ac-tubulin representing stable microtubules and increased expression of Tyr-tubulin representing unstable microtubules, respectively, compared with control cells (Fig. 5g, h). Remarkably, the effects of glucolipotoxicity were reversed when GSK3β was inactivated (Fig. 5b-h; Additional file 1: Figure. S8b,c).

### Mitochondrial transport and function are impaired by glucolipotoxicity-activated GSK3β in RGCs

**Fig. 6:**
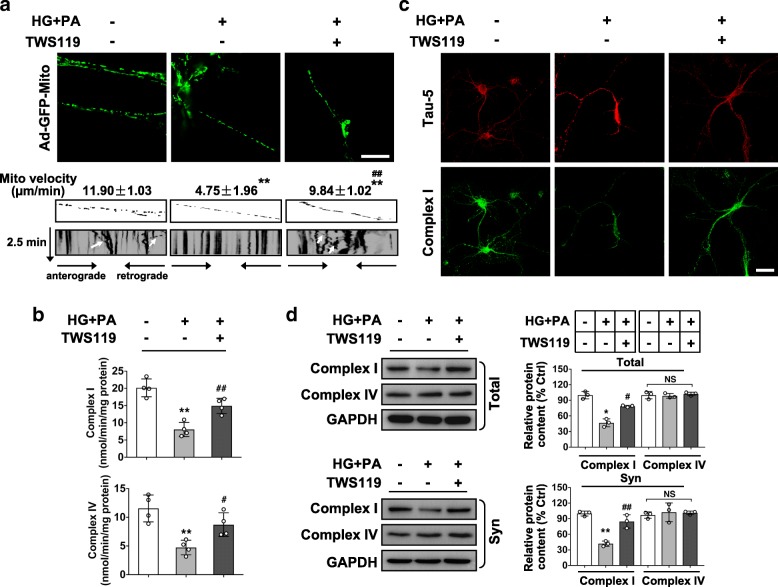
Mitochondrial transport and function are impaired at glucolipotoxicity-stressed RGCs in a GSK3β-dependent manner. (**a**) Mitochondrial axonal trafficking was determined by infecting RGCs with an adenovirus vector carrying a foreign gene for mitochondrial complex IV (Ad-GFP-Mito). Representative fluorescence images for GFP-labeled mitochondria within axons (upper panels) and kymograph images of axonal mitochondrial movement (middle and bottom panels) are shown. Traces of moving mitochondria are indicated with white arrow. The average transport speed of movable mitochondria was calculated and expressed as mito velocity (μm/min). (**b**) Activity of mitochondrial complex I and complex IV was measured by spectrophotometry and expressed as nmol/min/mg protein. (**c**) Representative images of double immunofluoresence for Tau 5 (red) and complex I (green). Scale bar, 40 μm. (**d**) Western blotting for complex I and complex IV from whole cell lysates (Total) or synaptosome fractions (Syn). Intensities were quantified and normalized against the level of GAPDH and expressed as percentage of protein abundance under stimulation relative to control. Data are means ± SEM of three (**a**, **d**) or four (**b**) independent experiments. ^*^P < 0.05 and ^**^P < 0.01 vs control; ^#^*P* < 0.05 and ^##^*P* < 0.01 vs HG + PA. NS, no significant difference

Destabilized microtubule track by glucolipotoxicity might retard long distance transport of mitochondria to presynaptic sites to fulfill their synaptic functions. Here, we monitored mitochondrial trafficking within axonal processes of RGCs infected with an adenoviral vector Ad-GFP-mito. RGCs under glucolipotoxic stress displayed significantly decreased mitochondrial mobility, relative to control cells (Fig. 6a). We further determined whether glucolipotoxic stress affects mitochondrial function by examining the activity and expression of NADH-ubiquinone oxidoreductase (complex I), the first complex in the electron transport chain (ETC), and cytochrome-c oxidase complex IV, catalyzing the final step in the ETC for ATP synthesis. Glucolipotoxicity significantly impaired the activity of complex I and IV (Fig. 6b), reduced complex I expression in whole cell lysates and synaptosome, but barely changed the expression of complex IV (Fig. 6d). Immunostaining also revealed the deprivation of functional mitochondrial complex I in particular from the neurites of RGCs upon stress (Fig. 6c). Thus, the reduced mitochondrial trafficking and activity by glucolipotoxicity may lead to short of energy production through mitochondria in synapses, which was rescued by GSK3β inhibition.

### Tau depletion rescues glucolipotoxicity-induced mitochondrial abnormalities and presynaptic loss in RGCs

**Fig. 7:**
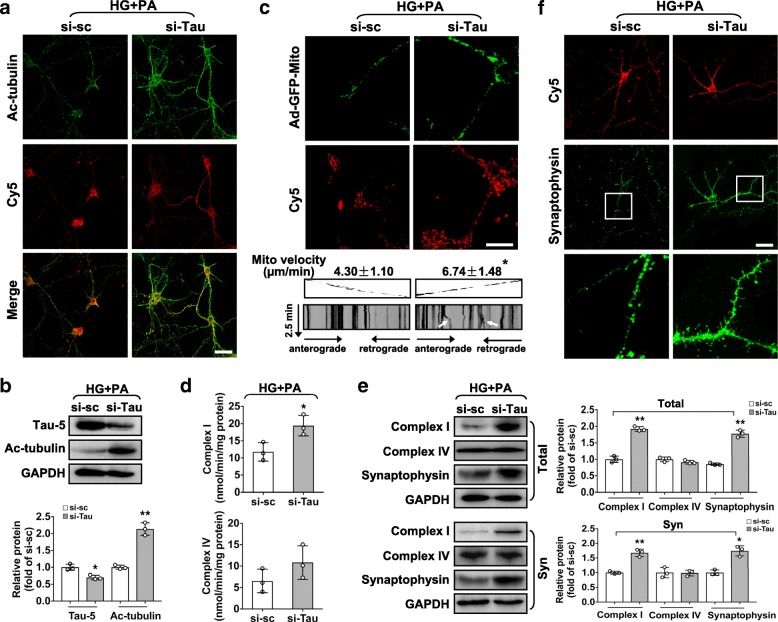
Knock-down of tau rescues mitochondrial abnormalities and presynaptic loss in glucolipotoxicity-stressed RGCs**.** RGCs were transfected with a Cy5-labeled siRNA (si-Tau or si-sc) and treated with HG + PA. (**a**) Representative immunocytochemistry for Ac-tubulin (green, Ac-tubulin; red, Cy5; scale bar, 40 μm). (**b**) Western blotting for Ac-tubulin and Tau 5. Intensities were normalized against the level of GAPDH and expressed as fold changes of protein abundance with si-Tau relative to si-sc control. (**c**) Mitochondrial transport in siRNA-transfected RGCs was performed by infecting cultured RGCs with Ad-GFP-Mito and treated with HG + PA. Representative fluorescence images (upper panels; scale bar, 10 μm) for GFP-labeled mitochondria (GFP, green) within axons in siRNA-transfected cells (Cy5, red) and kymograph images of axonal mitochondrial movement (middle and bottom panels) are shown. Traces of moving mitochondria are indicated with white arrow. The average transport speed of movable mitochondria was calculated. (**d**) Activity of mitochondrial complex I and complex IV. (**e**) Western blotting for complex I, complex IV and synaptophysin from whole cell lysates (Total) or synaptosome fractions (Syn). Intensities were normalized against the level of GAPDH and expressed as fold changes of protein abundance with si-Tau relative to si-sc control. (**f**) Representative fluorescence images for synaptophysin (green) in siRNA-transfected cells (Cy5, red). Areas boxed in for synaptophysin immunostaining are shown at higher magnification. Scale bar, 40 μm. Data are means ± SEM of three independent experiments. ^*^P < 0.05 and ^**^P < 0.01 vs control si-sc

To further evaluate the role of tau upon glucolipotoxicity, knock-down of tau in RGCs was performed by transfection of Cy5-labeled si-Tau. Tau repression was confirmed by reduced levels of total and phosphorylated tau (Additional file 1: Figure. S9a). Knock-down of tau with si-Tau led to considerably improved microtubule stability (Fig. 7a,b), mitochondrial mobility (Fig. 7c) and activity (Fig. 7d,e; Additional file 1: Figure. S9b), thus restored the expression of synaptophysin (Fig. 7e,f) in glucolipotoxicity-stressed RGCs, as compared to si-sc control.

## Discussion

Combining clinically used electrophysiological techniques and molecular analyses in vivo and in vitro, the present study reveals for the first time that hyperphosphorylated tau plays a toxic role in the context of HFD-induced diabetic RGCs neurodegeneration. First, using a well characterized HFD-induced mouse diabetes model, we show that visual dysfunction of RGCs in the absence of detectable microvasculopathy are closely associated with synapse and axon degeneration starting well before the apoptosis of RGCs. Second, we demonstrate that increased tau phosphorylation contributes to synapse loss and reduced activity of RGCs since selected knock-down of tau remarkably reverses the neurodegenerative phenotype and RGCs dysfunction in the HFD-induced DR model. Third, activation of GSK3β by impaired IRS1/Akt signaling is required for HFD-induced tau hyperphosphorylation, resulting in RGCs dysfunction and synapse loss in HFD-induced diabetic retinae. Last, in vitro data demonstrate a direct role for hyperphosphorylated tau in glucolipotoxicity-induced RGCs synapse loss, through impairing mitochondrial trafficking and reducing mitochondrial activity in a GSK3β-dependent manner. Based on these observations, we thus propose mild retinal tauopathy as a new pathophysiological model for DR, which is characterized by increased tau phosphorylation leading to RGCs neurodgeneration.

Even though a number of investigations have suggested that thinning of the inner retina due to apoptosis and loss of RGCs is associated with visual impairment in rodent models of type 2 diabetes [7, 35], our data revealed that HFD-induced electrophysiological impairment of RGCs, including delayed and diminished visual responses to patterned light stimuli, precedes significant apoptosis of RGCs. These observations suggest that apoptosis/loss of RGCs is not a prerequisite for visual dysfunction in early phase of DR. The discrepancies about the time required for induction of RGCs apoptosis in studies of experimental diabetes may be explained by differences in strains and disease models. Nonetheless, the absence of significant apoptosis of RGCs at the early time point of feeding regimen in this study is in consistent with a recent work where only a few apoptotic RGCs are detected in the GCL from rats fed with HFD for 12 weeks, despite a substantially increased apoptosis in the outer and inner nuclear layer of retinae [22].

In contrast to RGCs apoptosis, considerable loss of presynaptic protein, synaptophysin, was found in mice fed with HFD for as early as 20 weeks, a time when compromised activity of RGCs was triggered. Loss of synaptophysin has been correlated with impaired synaptic plasticity and electrophysiology, probably due to reduced neurotransmitter release and vesicle recycling in diabetic rat hippocampus [36, 37]. Likewise, synaptophysin depletion from retinae by HFD intake may contribute to functional deficits of RGCs, most likely by affecting the retinal presynaptic neurotransmitter vesicle pool in a similar manner as that in brains from diabetic rats. Moreover, the present results suggest a progression of RGCs degeneration in HFD-induced type 2 diabetes, where synaptic decline and axon thinning precede neuronal apoptosis and are the basis for the observed visual deficits. Evidence of axon degeneration occurring before soma degeneration has also been reported in a streptozotocin (STZ)-induced DR model [9] and other neurodegenerative eye diseases, such as glaucoma [38]. Collectively, our findings demonstrate that reduced visual response of RGCs by HFD feeding is caused at least partially by synapse and axon degeneration starting well before the appearance of clear signs of RGCs apoptosis.

Although several lines of evidence have proposed a connection between tau hyperphosphorylation and synaptic degeneration in AD brains [39–41], the effect of tau in synaptic dysfunction, if any, in the context of diabetes-induced retinal neurodegeneration has yet to be ascertained. Here, we show for the first time that synaptic decline is triggered by the presence of abnormal hyperphosphorylation of tau at S396/S404 and T205/T231 in HFD-induced diabetic retinae, since selected depletion of tau by si-Tau or dephosphorylation of tau by GSK3β inhibition rescues synapse loss and vision deficits. Furthermore, we found that abnormal hyperphosphorylation of tau in primary RGCs by glucolipotoxicity mimicking the HFD-induced diabetes in vitro, led to dissociation of normal tau from tubulin, destabilization of microtubules and impairment of axonal mitochondrial trafficking. As a result, inhibition of microtubule-dependent trafficking of mitochondria may deprive synapses of mitochondria and cause the starvation of synapses, leading to synaptic dysfunction of glucolipotoxicity-stressed RGCs.

In addition,we found decreased protein levels of ETC complex I, but not complex IV, in total and synaptosome extract of cultured RGCs upon glucolipotoxic stress, indicating that hyperphosphorylated tau may impair acutely the initial step of energy production through mitochondria, rendering complex IV be affected in a later stage of pathology. Our in vivo data further confirmed markedly reduced complex I in the retina from mice fed with HFD for as early as 20 weeks (Additional file 1: Figure. S10a,b). Collectively, these results propose that in addition to affect the synaptic targeting of mitochondria, thus reducing numbers of presynaptic mitochondria, an alternative pathway by which phospho-tau affects synaptic function is the direct inhibition of energy production through the mitochondria in synapses, which consequently lead to further decreased anterograde and retrograde movement of microtubule-dependent cargoes, such as mitochondria, synaptic vesicles and mRNA transcripts.

It is worthy to note that in contrast to whole cell lysate, where synaptophysin protein loss precedes statistic decrease in mRNA transcripts, the reduction in protein levels of synaptophysin in synaptosomes is concomitant with a decline in their synaptic mRNA contents in the context of glucolipotoxicity-stressed RGCs. This finding suggests that the loss of synaptic protein is likely due to a downregulation of localized protein synthesis within synapses by multiple regulatory mechanisms. First, hyperphosphorylated tau may decrease axonal/dendritic transport of mRNA transcripts and translational machinery such as mitochondria, Golgi, and rough endoplasmic reticulum (RER), into distal synapses [42, 43] through disrupting microtubule tracks in RGCs, leading to compromised capacity for localized protein synthesis. Alternatively, in addition to inhibiting synaptic energy production via decreasing complex I, hyperphosphorylated tau might also induce alterations and a consequent reduction of Golgi and RER [44], thus jeopardizing synaptic protein synthesis.

To our knowledge, the present study also shows for the first time that the abnormal tau hyperphosphorylation in HFD-induced diabetic retinae represents a pathological target of dysregulated insulin signaling through GSK3β activation, similar as that in HFD-induced diabetic brains [17, 45]. The resulting loss of IRS-1 and Akt activity by HFD intake is predicted to activate GSK3β, a major tau kinase, leading to increased tau phosphorylation. This process may further dysregulate insulin signaling since GSK3β per se directly phosphorylates IRS-1 at Ser 322 to facilitate IRS-1 inactivation [46], thus aggravating tau pathology. In support of this hypothesis, we found that GSK3β inactivation by intraocular injection of its inhibitor TWS119 resulted in the rescue of mitochondrial dysfunction (Additional file 1: Fig. S10c), synapse loss and visual impairment in HFD-induced diabetes mouse models, in association with reduced tau phosphorylation in the retina. Indeed, reducing tau hyperphosphorylation through a specific kinase (e.g. GSK3β) inhibition has emerged as a therapeutic target for drug development for tau pathology in neurodegenerative disease [47]. However, one cannot exclude the possibility that retinal GSK3β inhibition may also activate its downstream β-catenin signaling, the sustained activation of which can further lead to the development of retinal vascular abnormalities [48, 49]. In such case, a cell-type (retinal neuron) specific targeting of GSK3β would be highly desirable to avoid the off-target effect of a kinase inhibitor.

A recent study has demonstrated that reducing tau levels and phosphorylation by intraocular injection of tau-specific siRNA can protect RGCs from hypertension-induced damage in a rodent model of glaucoma [33], making it a promising strategy to limit the toxic effects of tau. Using this siRNA-based strategy, we show that intravitreous administration of si-Tau reduced retinal levels of phosphorylated and total tau, promoted robust expression of synaptophysin and complex I (Additional file 1: Figure S10d), and improved visual function of RGCs in HFD-induced DR eyes. Our data thus provide strong proof of principle for a detrimental gain-of-function role of hyperphosphorylated tau in DR. Of interest, although mice accumulated hyperphosphorylated tau at 16 weeks after HFD, they did not develop synapse loss and visual deficits of RGCs until 20 weeks after HFD, suggesting that the impaired RGCs activity seems directly related to reaching a certain threshold of tau pathology in this context. Thus, the onset of si-Tau treatment at 20 weeks after HFD was selected to evaluate the feasibility of selected tau depletion for the treatment of retinal neurodegeneration in DR. Our finding that si-Tau effectively rescued RGCs by ameliorating tau pathology and synapse loss also indicates that the toxic effects of tau on synapses may be reversible within a certain time frame. Considering that sublethal impairment of RGCs synapses could result in progressive dying back toward the cell body, which cannot be reversed once developed, rescue of synaptic loss via knock-down of tau in the retina at a time when RGCs apoptosis not yet occurs appears to have a widespread beneficial effect on the overall health of RGCs, leading to remarkable improvements in visual functions impaired by HFD-induced diabetes.

## Conclusions

In summary, combining clinically used electrophysiological techniques and molecular analyses in vivo and in vitro, this study identifies hyperphosphorylated tau as a toxic mediator in synapse loss and visual dysfunction of RGCs in HFD-induced diabetic retinae via GSK3β activation. We also develop a new therapeutic strategy in which intravitreal injection of a siRNA specific for tau or an inhibitor of GSK3β reverses synapse loss and visual dysfunction of RGCs by attenuating tau hyperphosphorylation at the onset of DR. In addition, our data highlight the potential cellular mechanisms by which hyperphosphorylated tau induces synaptic loss and dysfunction, including i) inhibition of localized synaptic protein synthesis by impairing microtubule-dependent trafficking of translational machineries; ii) disruption of the synaptic roles of mitochondria by reducing its energy production. Overall, these results propose mild retinal tauopathy as a new pathophysiological model for DR and open new avenues for neuroprotective intervention strategies of DR by targeting of tau to counter RGCs neurodegeneration occurring before retinal vasculature abnormalities.

## Additional file

Additional file 1: **Figure S1.** Synapse loss occurs before apoptosis in RGCs from HFD-induced type 2 diabetic mice. (a-g) HFD-fed mice developed features of type 2 diabetes. (h-i) Western blot. (j) Evans Blue angiography. n = 6 (a-g) or = 4 (h-i) animals per group. *P < 0.05 and **P < 0.01 vs age-match RD controls. **Figure S2.** HFD promotes hyperphosphorylation of tau in neural retina and optic nerves. Double immunostaining in retina (a) or in RGCs axons (b). **Figure S3.** Knock-down of tau does not cause disease phenotype under physiological conditions. (a) Knock-down efficiency of a targeted si-Tau. n = 3 eyes per group. *P < 0.05 vs si-sc. (b-c) Immunostaining. **Figure S4.** Intravitreal delivery of si-Tau prevents RGCs from HFD-induced tau phosphorylation and synapse loss. (a-c) Immunostaining. *P < 0.05 and **P < 0.01 vs si-sc. **Figure S5.** Intravitreal injection of a GSK3β inhibitor TWS119 reduces retinal tau hyperphosphorylation and synapse loss in HFD-fed mice. (a-c) Immunostaining. *P < 0.05 and **P < 0.01 vs contralateral eye injected with vehicle. **Figure S6.** Intravitreal delivery of si-GSK3β protects RGCs from HFD-induced vision and synapse loss. (a) Representative VEP. (b) Knock-down efficiency of targeted si-GSK3β. (c-e) Representative immunostaining. n = 4 eyes per group. **P < 0.01 vs si-sc. **Figure S7.** Inhibition of GSK3β by TWS119 does not trigger disease phenotype under physiological conditions. (a) VEP. (b-c) Immunostaining. **Figure S8.** Glucolipotoxicity induces dysregulation of Akt/GSK3β signaling and tau hyperphosphorylation in primary RGCs. (a-b) Western blot. (c) Immunostaining. *P < 0.05 and **P < 0.01 vs control; #*P* < 0.05 and ##*P* < 0.01 vs HG + PA. **Figure S9.** Selected tau depletion by siRNA prevents RGCs from glucolipotoxicity-induced mitochondria dysfunction. (a) Western blot. (b) Immunostaining. *P < 0.05 and **P < 0.01 vs si-sc. **Figure S10.** Tau depletion or GSK3β inhibition restores HFD-impaired mitochondrail activity. (a) Western blot. (b-d) Retinal double immunostaining. n = 4 (a), = 6 (b), or = 5 (c-d) mice per group. **P < 0.01 vs age-match RD controls. (ZIP 7945 kb)

## Abbreviations

AD: Alzheimer’s disease
DR: diabetic retinopathy
EB: Evans Blue
ERG: electroretinogram
FFA: fundus fluorescein angiography
GCL: ganglion cell layer
GSK3β: glycogen synthase kinase 3β
HFD: high-fat diet
INL: inner nuclear layer
IPL: inner plexiform layer
OPL: outer plexiform layer
PERG: pattern ERG
Q-PCR: quantitative real-time polymerase chain reaction
RD: regular standard laboratory chow
RER: rough endoplasmic reticulum
RGCs: retinal ganglion cells
siRNA: short interfering RNA
STZ: streptozotocin
TG: triglyceride
VEP: visual evoked potential
