# Supplementary figures and images for: GSK3β-mediated tau hyperphosphorylation triggers diabetic retinal neurodegeneration by disrupting synaptic and mitochondrial functions
Source: Mol Neurodegener. 2018 Nov 22;13:62. doi: 10.1186/s13024-018-0295-z (PMC6251088; doi:10.1186/s13024-018-0295-z)

Suppl. Figure 2

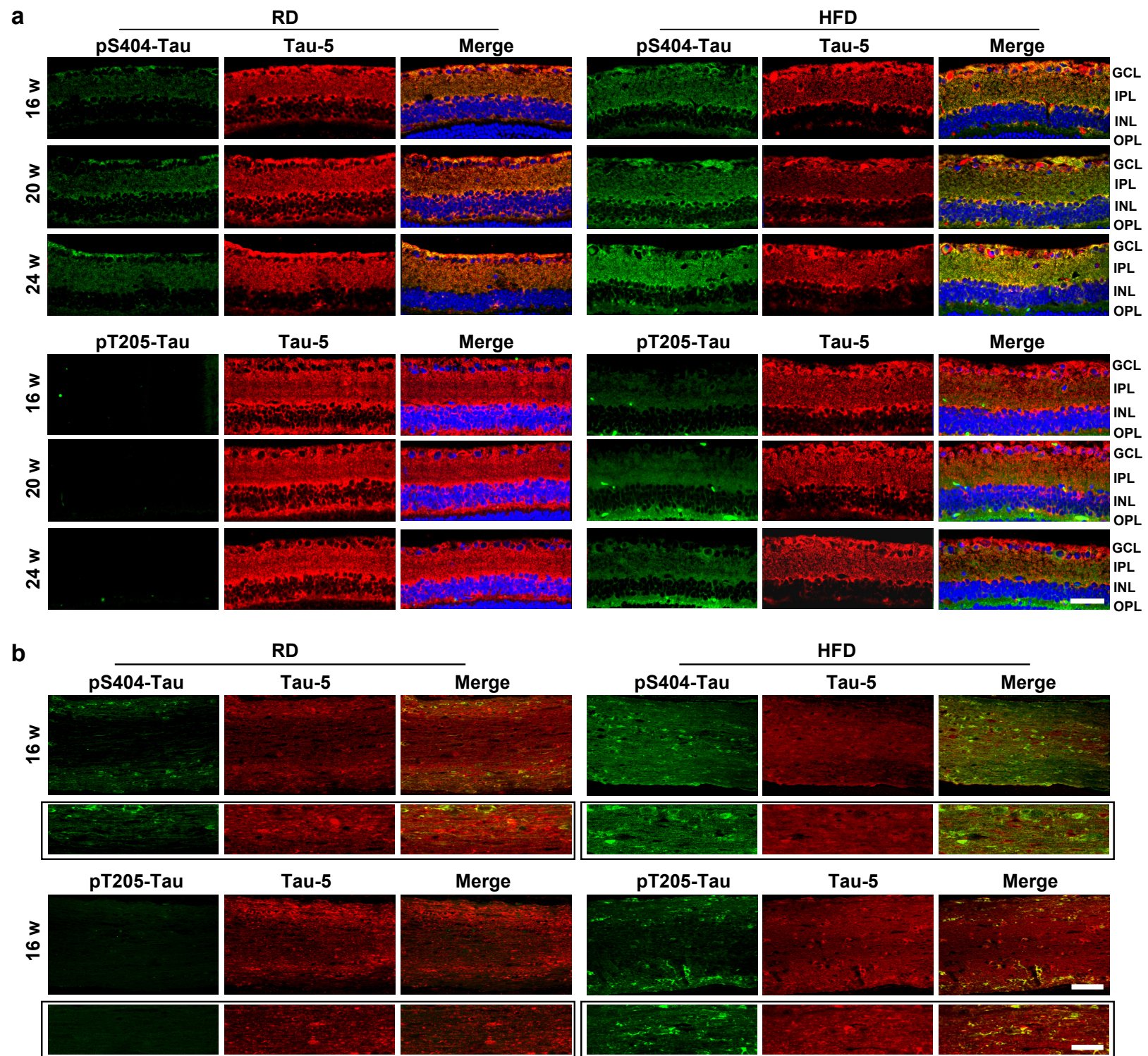

Supplement: Supplementary file 1 — Figure S1. Synapse loss occurs before apoptosis in RGCs from HFD-induced type 2 diabetic mice. (a-g) HFD-fed mice developed features of type 2 diabetes. (h-i) Western blot. (j) Evans Blue angiography. n = 6 (a-g) or = 4 (h-i) animals per group. *P < 0.05 and **P < 0.01 vs age-match RD controls. Figure S2. HFD promotes hyperphosphorylation of tau in neural retina and optic nerves. Double immunostaining in retina (a) or in RGCs axons (b). Figure S3. Knock-down of tau does not cause disease phenotype under physiological conditions. (a) Knock-down efficiency of a targeted si-Tau. n = 3 eyes per group. *P < 0.05 vs si-sc. (b-c) Immunostaining. Figure S4. Intravitreal delivery of si-Tau prevents RGCs from HFD-induced tau phosphorylation and synapse loss. (a-c) Immunostaining. *P < 0.05 and **P < 0.01 vs si-sc. Figure S5. Intravitreal injection of a GSK3β inhibitor TWS119 reduces retinal tau hyperphosphorylation and synapse loss in HFD-fed mice. (a-c) Immunostaining. *P < 0.05 and **P < 0.01 vs contralateral eye injected with vehicle. Figure S6. Intravitreal delivery of si-GSK3β protects RGCs from HFD-induced vision and synapse loss. (a) Representative VEP. (b) Knock-down efficiency of targeted si-GSK3β. (c-e) Representative immunostaining. n = 4 eyes per group. **P < 0.01 vs si-sc. Figure S7. Inhibition of GSK3β by TWS119 does not trigger disease phenotype under physiological conditions. (a) VEP. (b-c) Immunostaining. Figure S8. Glucolipotoxicity induces dysregulation of Akt/GSK3β signaling and tau hyperphosphorylation in primary RGCs. (a-b) Western blot. (c) Immunostaining. *P < 0.05 and **P < 0.01 vs control; #P < 0.05 and ##P < 0.01 vs HG + PA. Figure S9. Selected tau depletion by siRNA prevents RGCs from glucolipotoxicity-induced mitochondria dysfunction. (a) Western blot. (b) Immunostaining. *P < 0.05 and **P < 0.01 vs si-sc. Figure S10. Tau depletion or GSK3β inhibition restores HFD-impaired mitochondrail activity. (a) Western blot. (b-d) Retinal double immunostaini [file 13024_2018_295_MOESM1_ESM.zip › Fig.S2.pdf]

Suppl. Figure 10

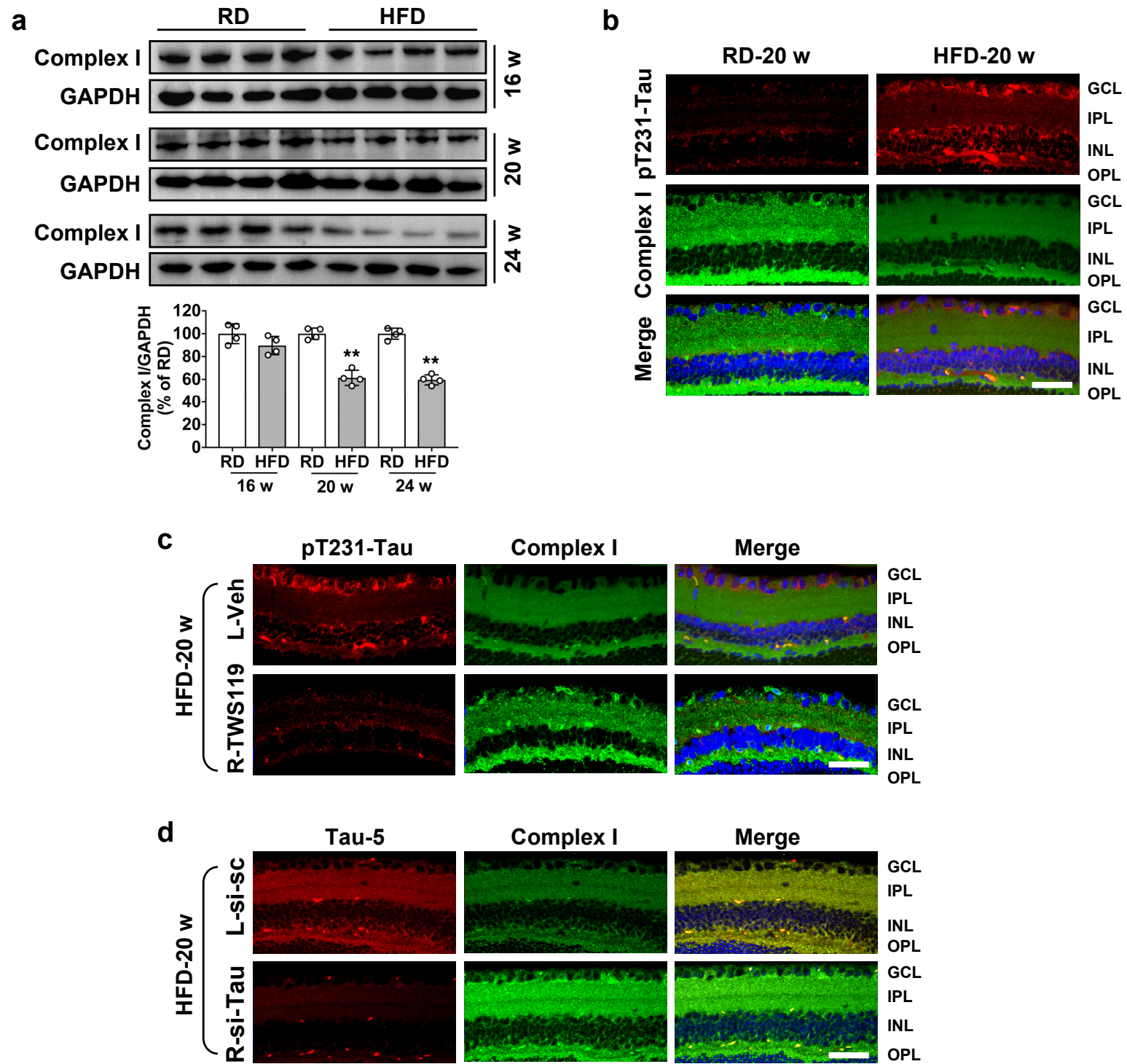

Supplement: Supplementary file 1 — Figure S1. Synapse loss occurs before apoptosis in RGCs from HFD-induced type 2 diabetic mice. (a-g) HFD-fed mice developed features of type 2 diabetes. (h-i) Western blot. (j) Evans Blue angiography. n = 6 (a-g) or = 4 (h-i) animals per group. *P < 0.05 and **P < 0.01 vs age-match RD controls. Figure S2. HFD promotes hyperphosphorylation of tau in neural retina and optic nerves. Double immunostaining in retina (a) or in RGCs axons (b). Figure S3. Knock-down of tau does not cause disease phenotype under physiological conditions. (a) Knock-down efficiency of a targeted si-Tau. n = 3 eyes per group. *P < 0.05 vs si-sc. (b-c) Immunostaining. Figure S4. Intravitreal delivery of si-Tau prevents RGCs from HFD-induced tau phosphorylation and synapse loss. (a-c) Immunostaining. *P < 0.05 and **P < 0.01 vs si-sc. Figure S5. Intravitreal injection of a GSK3β inhibitor TWS119 reduces retinal tau hyperphosphorylation and synapse loss in HFD-fed mice. (a-c) Immunostaining. *P < 0.05 and **P < 0.01 vs contralateral eye injected with vehicle. Figure S6. Intravitreal delivery of si-GSK3β protects RGCs from HFD-induced vision and synapse loss. (a) Representative VEP. (b) Knock-down efficiency of targeted si-GSK3β. (c-e) Representative immunostaining. n = 4 eyes per group. **P < 0.01 vs si-sc. Figure S7. Inhibition of GSK3β by TWS119 does not trigger disease phenotype under physiological conditions. (a) VEP. (b-c) Immunostaining. Figure S8. Glucolipotoxicity induces dysregulation of Akt/GSK3β signaling and tau hyperphosphorylation in primary RGCs. (a-b) Western blot. (c) Immunostaining. *P < 0.05 and **P < 0.01 vs control; #P < 0.05 and ##P < 0.01 vs HG + PA. Figure S9. Selected tau depletion by siRNA prevents RGCs from glucolipotoxicity-induced mitochondria dysfunction. (a) Western blot. (b) Immunostaining. *P < 0.05 and **P < 0.01 vs si-sc. Figure S10. Tau depletion or GSK3β inhibition restores HFD-impaired mitochondrail activity. (a) Western blot. (b-d) Retinal double immunostaini [file 13024_2018_295_MOESM1_ESM.zip › Suppl Fig.10-revision.pdf]

Suppl. Figure 1

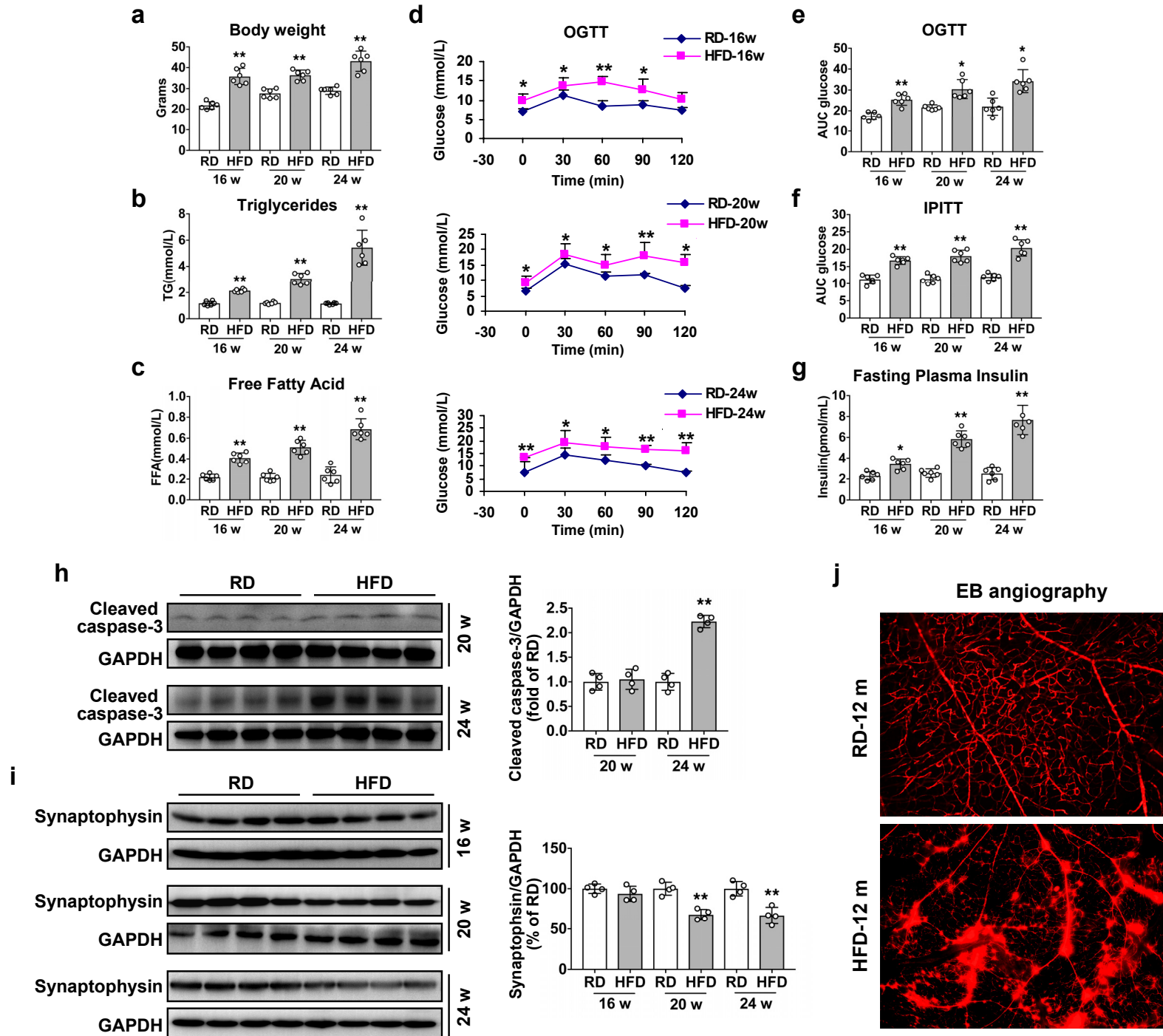

Supplement: Supplementary file 1 — Figure S1. Synapse loss occurs before apoptosis in RGCs from HFD-induced type 2 diabetic mice. (a-g) HFD-fed mice developed features of type 2 diabetes. (h-i) Western blot. (j) Evans Blue angiography. n = 6 (a-g) or = 4 (h-i) animals per group. *P < 0.05 and **P < 0.01 vs age-match RD controls. Figure S2. HFD promotes hyperphosphorylation of tau in neural retina and optic nerves. Double immunostaining in retina (a) or in RGCs axons (b). Figure S3. Knock-down of tau does not cause disease phenotype under physiological conditions. (a) Knock-down efficiency of a targeted si-Tau. n = 3 eyes per group. *P < 0.05 vs si-sc. (b-c) Immunostaining. Figure S4. Intravitreal delivery of si-Tau prevents RGCs from HFD-induced tau phosphorylation and synapse loss. (a-c) Immunostaining. *P < 0.05 and **P < 0.01 vs si-sc. Figure S5. Intravitreal injection of a GSK3β inhibitor TWS119 reduces retinal tau hyperphosphorylation and synapse loss in HFD-fed mice. (a-c) Immunostaining. *P < 0.05 and **P < 0.01 vs contralateral eye injected with vehicle. Figure S6. Intravitreal delivery of si-GSK3β protects RGCs from HFD-induced vision and synapse loss. (a) Representative VEP. (b) Knock-down efficiency of targeted si-GSK3β. (c-e) Representative immunostaining. n = 4 eyes per group. **P < 0.01 vs si-sc. Figure S7. Inhibition of GSK3β by TWS119 does not trigger disease phenotype under physiological conditions. (a) VEP. (b-c) Immunostaining. Figure S8. Glucolipotoxicity induces dysregulation of Akt/GSK3β signaling and tau hyperphosphorylation in primary RGCs. (a-b) Western blot. (c) Immunostaining. *P < 0.05 and **P < 0.01 vs control; #P < 0.05 and ##P < 0.01 vs HG + PA. Figure S9. Selected tau depletion by siRNA prevents RGCs from glucolipotoxicity-induced mitochondria dysfunction. (a) Western blot. (b) Immunostaining. *P < 0.05 and **P < 0.01 vs si-sc. Figure S10. Tau depletion or GSK3β inhibition restores HFD-impaired mitochondrail activity. (a) Western blot. (b-d) Retinal double immunostaini [file 13024_2018_295_MOESM1_ESM.zip › Suppl Fig.1-revision.pdf]

# Suppl. Figure 3

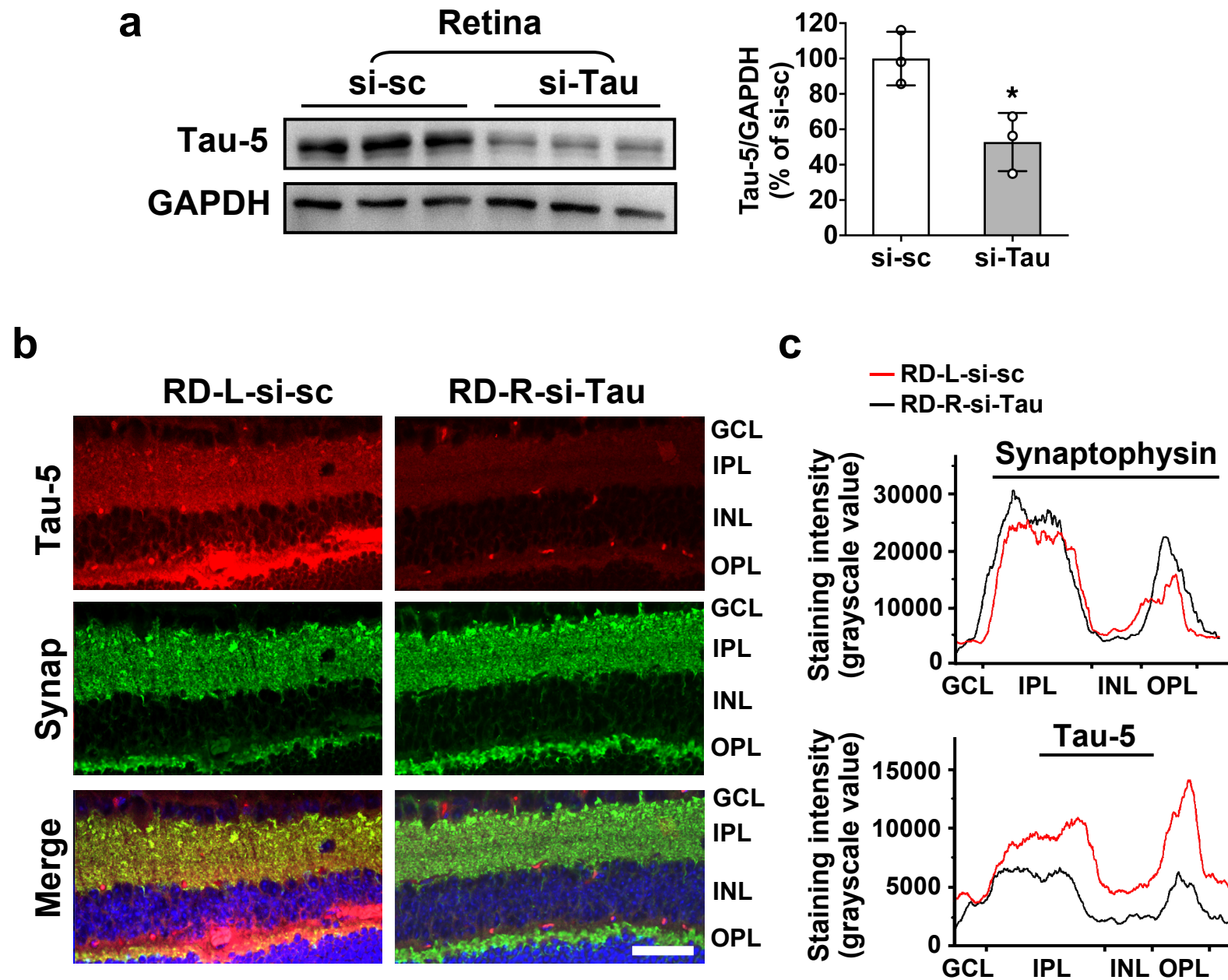

Supplement: Supplementary file 1 — Figure S1. Synapse loss occurs before apoptosis in RGCs from HFD-induced type 2 diabetic mice. (a-g) HFD-fed mice developed features of type 2 diabetes. (h-i) Western blot. (j) Evans Blue angiography. n = 6 (a-g) or = 4 (h-i) animals per group. *P < 0.05 and **P < 0.01 vs age-match RD controls. Figure S2. HFD promotes hyperphosphorylation of tau in neural retina and optic nerves. Double immunostaining in retina (a) or in RGCs axons (b). Figure S3. Knock-down of tau does not cause disease phenotype under physiological conditions. (a) Knock-down efficiency of a targeted si-Tau. n = 3 eyes per group. *P < 0.05 vs si-sc. (b-c) Immunostaining. Figure S4. Intravitreal delivery of si-Tau prevents RGCs from HFD-induced tau phosphorylation and synapse loss. (a-c) Immunostaining. *P < 0.05 and **P < 0.01 vs si-sc. Figure S5. Intravitreal injection of a GSK3β inhibitor TWS119 reduces retinal tau hyperphosphorylation and synapse loss in HFD-fed mice. (a-c) Immunostaining. *P < 0.05 and **P < 0.01 vs contralateral eye injected with vehicle. Figure S6. Intravitreal delivery of si-GSK3β protects RGCs from HFD-induced vision and synapse loss. (a) Representative VEP. (b) Knock-down efficiency of targeted si-GSK3β. (c-e) Representative immunostaining. n = 4 eyes per group. **P < 0.01 vs si-sc. Figure S7. Inhibition of GSK3β by TWS119 does not trigger disease phenotype under physiological conditions. (a) VEP. (b-c) Immunostaining. Figure S8. Glucolipotoxicity induces dysregulation of Akt/GSK3β signaling and tau hyperphosphorylation in primary RGCs. (a-b) Western blot. (c) Immunostaining. *P < 0.05 and **P < 0.01 vs control; #P < 0.05 and ##P < 0.01 vs HG + PA. Figure S9. Selected tau depletion by siRNA prevents RGCs from glucolipotoxicity-induced mitochondria dysfunction. (a) Western blot. (b) Immunostaining. *P < 0.05 and **P < 0.01 vs si-sc. Figure S10. Tau depletion or GSK3β inhibition restores HFD-impaired mitochondrail activity. (a) Western blot. (b-d) Retinal double immunostaini [file 13024_2018_295_MOESM1_ESM.zip › Suppl Fig.3-revision.pdf]

# Suppl. Figure 4

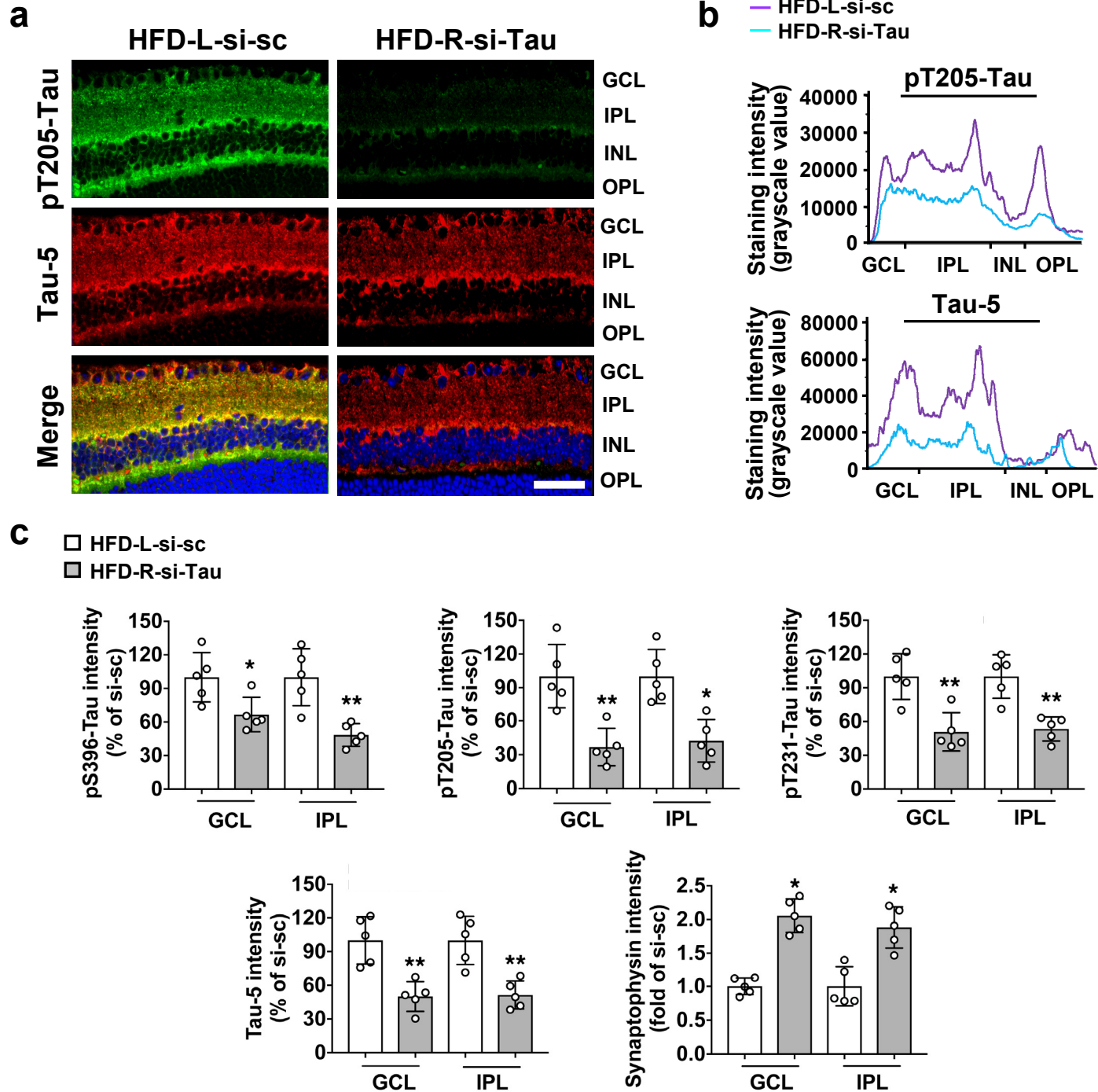

Supplement: Supplementary file 1 — Figure S1. Synapse loss occurs before apoptosis in RGCs from HFD-induced type 2 diabetic mice. (a-g) HFD-fed mice developed features of type 2 diabetes. (h-i) Western blot. (j) Evans Blue angiography. n = 6 (a-g) or = 4 (h-i) animals per group. *P < 0.05 and **P < 0.01 vs age-match RD controls. Figure S2. HFD promotes hyperphosphorylation of tau in neural retina and optic nerves. Double immunostaining in retina (a) or in RGCs axons (b). Figure S3. Knock-down of tau does not cause disease phenotype under physiological conditions. (a) Knock-down efficiency of a targeted si-Tau. n = 3 eyes per group. *P < 0.05 vs si-sc. (b-c) Immunostaining. Figure S4. Intravitreal delivery of si-Tau prevents RGCs from HFD-induced tau phosphorylation and synapse loss. (a-c) Immunostaining. *P < 0.05 and **P < 0.01 vs si-sc. Figure S5. Intravitreal injection of a GSK3β inhibitor TWS119 reduces retinal tau hyperphosphorylation and synapse loss in HFD-fed mice. (a-c) Immunostaining. *P < 0.05 and **P < 0.01 vs contralateral eye injected with vehicle. Figure S6. Intravitreal delivery of si-GSK3β protects RGCs from HFD-induced vision and synapse loss. (a) Representative VEP. (b) Knock-down efficiency of targeted si-GSK3β. (c-e) Representative immunostaining. n = 4 eyes per group. **P < 0.01 vs si-sc. Figure S7. Inhibition of GSK3β by TWS119 does not trigger disease phenotype under physiological conditions. (a) VEP. (b-c) Immunostaining. Figure S8. Glucolipotoxicity induces dysregulation of Akt/GSK3β signaling and tau hyperphosphorylation in primary RGCs. (a-b) Western blot. (c) Immunostaining. *P < 0.05 and **P < 0.01 vs control; #P < 0.05 and ##P < 0.01 vs HG + PA. Figure S9. Selected tau depletion by siRNA prevents RGCs from glucolipotoxicity-induced mitochondria dysfunction. (a) Western blot. (b) Immunostaining. *P < 0.05 and **P < 0.01 vs si-sc. Figure S10. Tau depletion or GSK3β inhibition restores HFD-impaired mitochondrail activity. (a) Western blot. (b-d) Retinal double immunostaini [file 13024_2018_295_MOESM1_ESM.zip › Suppl Fig.4-revision.pdf]

# Suppl. Figure 5

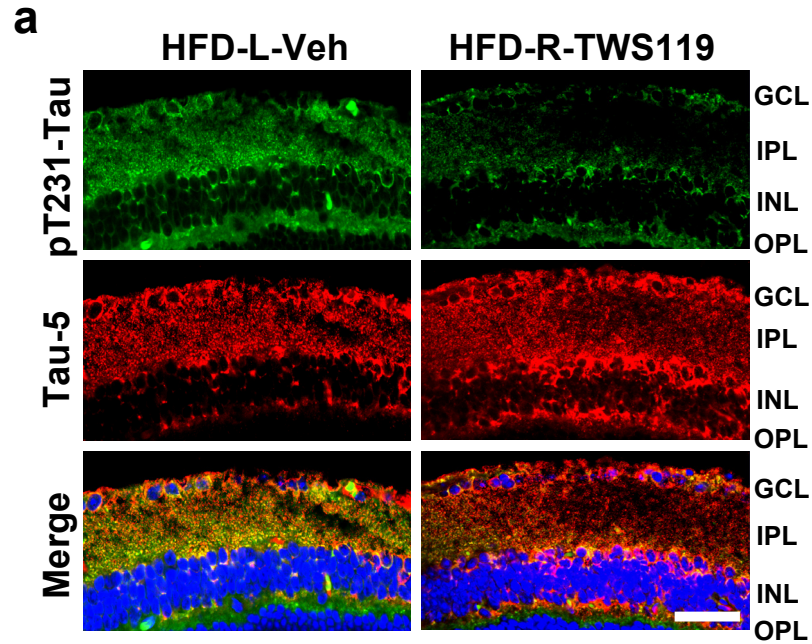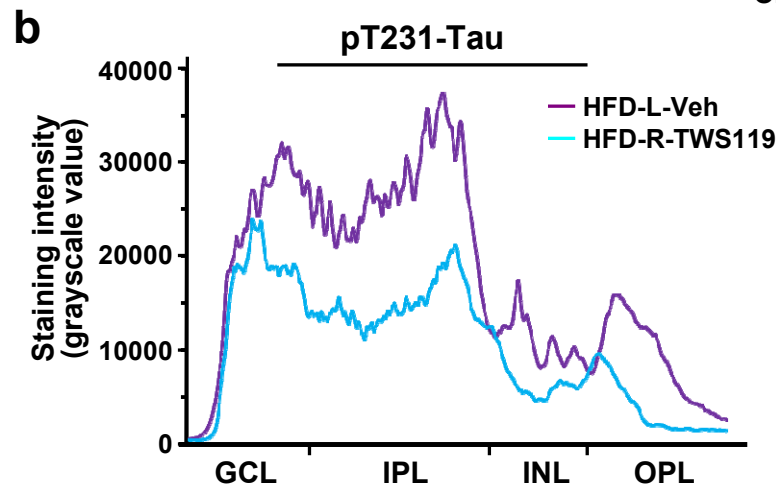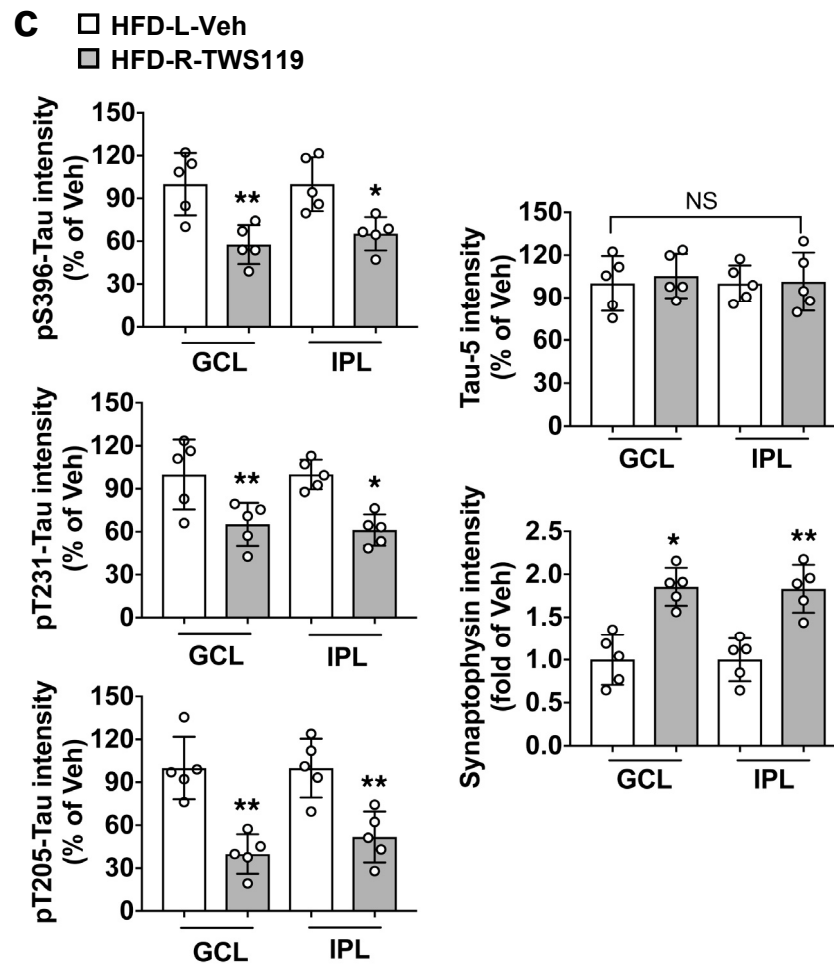

Supplement: Supplementary file 1 — Figure S1. Synapse loss occurs before apoptosis in RGCs from HFD-induced type 2 diabetic mice. (a-g) HFD-fed mice developed features of type 2 diabetes. (h-i) Western blot. (j) Evans Blue angiography. n = 6 (a-g) or = 4 (h-i) animals per group. *P < 0.05 and **P < 0.01 vs age-match RD controls. Figure S2. HFD promotes hyperphosphorylation of tau in neural retina and optic nerves. Double immunostaining in retina (a) or in RGCs axons (b). Figure S3. Knock-down of tau does not cause disease phenotype under physiological conditions. (a) Knock-down efficiency of a targeted si-Tau. n = 3 eyes per group. *P < 0.05 vs si-sc. (b-c) Immunostaining. Figure S4. Intravitreal delivery of si-Tau prevents RGCs from HFD-induced tau phosphorylation and synapse loss. (a-c) Immunostaining. *P < 0.05 and **P < 0.01 vs si-sc. Figure S5. Intravitreal injection of a GSK3β inhibitor TWS119 reduces retinal tau hyperphosphorylation and synapse loss in HFD-fed mice. (a-c) Immunostaining. *P < 0.05 and **P < 0.01 vs contralateral eye injected with vehicle. Figure S6. Intravitreal delivery of si-GSK3β protects RGCs from HFD-induced vision and synapse loss. (a) Representative VEP. (b) Knock-down efficiency of targeted si-GSK3β. (c-e) Representative immunostaining. n = 4 eyes per group. **P < 0.01 vs si-sc. Figure S7. Inhibition of GSK3β by TWS119 does not trigger disease phenotype under physiological conditions. (a) VEP. (b-c) Immunostaining. Figure S8. Glucolipotoxicity induces dysregulation of Akt/GSK3β signaling and tau hyperphosphorylation in primary RGCs. (a-b) Western blot. (c) Immunostaining. *P < 0.05 and **P < 0.01 vs control; #P < 0.05 and ##P < 0.01 vs HG + PA. Figure S9. Selected tau depletion by siRNA prevents RGCs from glucolipotoxicity-induced mitochondria dysfunction. (a) Western blot. (b) Immunostaining. *P < 0.05 and **P < 0.01 vs si-sc. Figure S10. Tau depletion or GSK3β inhibition restores HFD-impaired mitochondrail activity. (a) Western blot. (b-d) Retinal double immunostaini [file 13024_2018_295_MOESM1_ESM.zip › Suppl Fig.5-revision.pdf]

Suppl. Figure 6

a

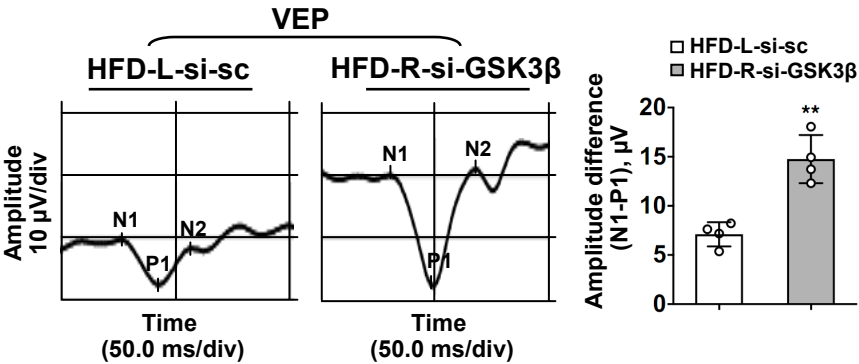

b

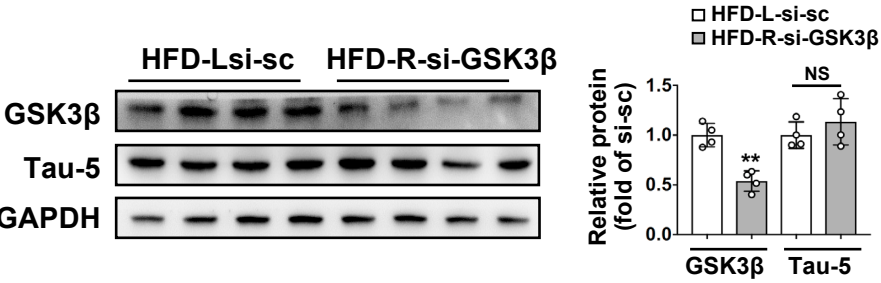

c

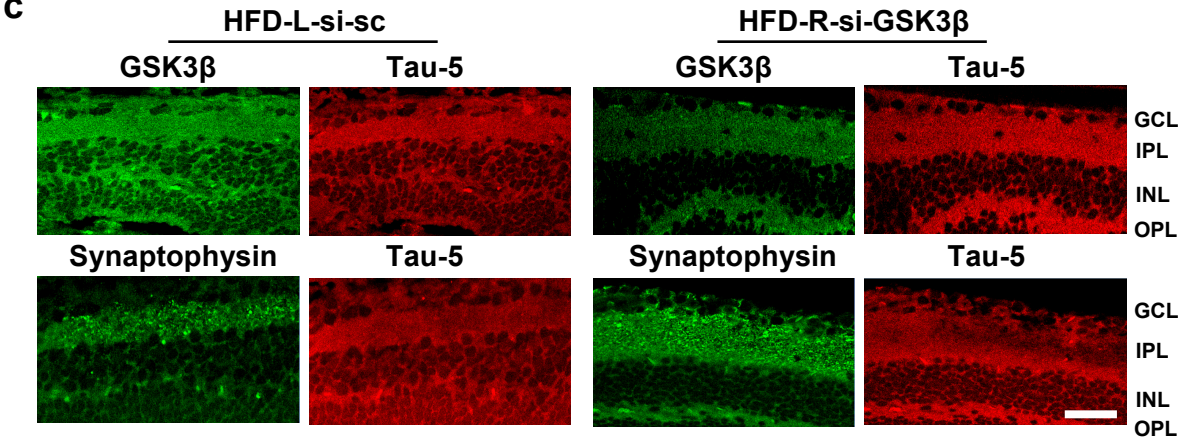

d

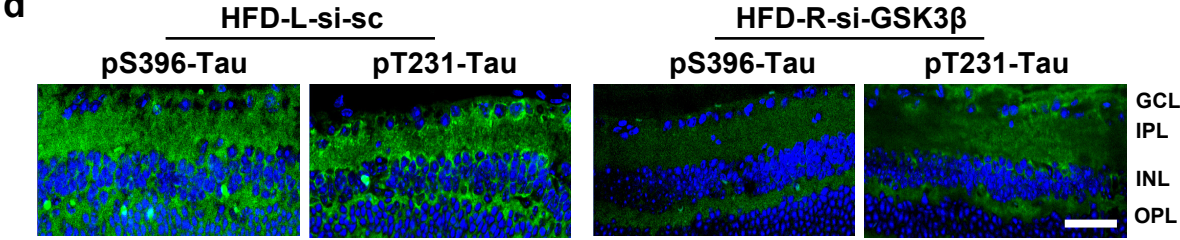

e

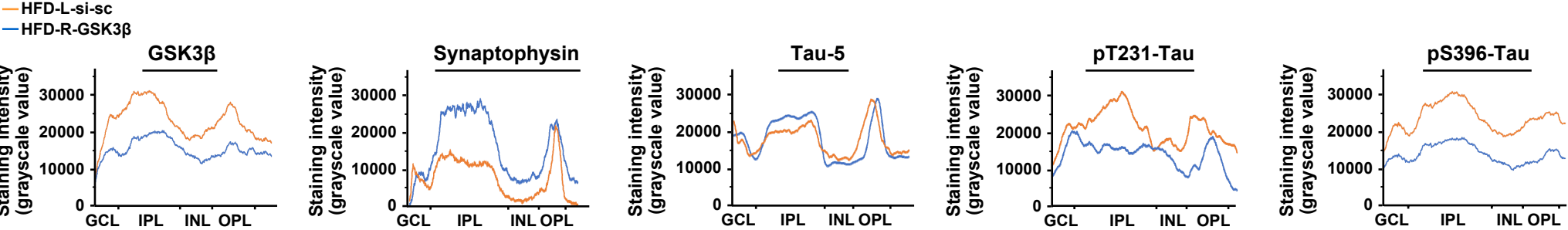

Supplement: Supplementary file 1 — Figure S1. Synapse loss occurs before apoptosis in RGCs from HFD-induced type 2 diabetic mice. (a-g) HFD-fed mice developed features of type 2 diabetes. (h-i) Western blot. (j) Evans Blue angiography. n = 6 (a-g) or = 4 (h-i) animals per group. *P < 0.05 and **P < 0.01 vs age-match RD controls. Figure S2. HFD promotes hyperphosphorylation of tau in neural retina and optic nerves. Double immunostaining in retina (a) or in RGCs axons (b). Figure S3. Knock-down of tau does not cause disease phenotype under physiological conditions. (a) Knock-down efficiency of a targeted si-Tau. n = 3 eyes per group. *P < 0.05 vs si-sc. (b-c) Immunostaining. Figure S4. Intravitreal delivery of si-Tau prevents RGCs from HFD-induced tau phosphorylation and synapse loss. (a-c) Immunostaining. *P < 0.05 and **P < 0.01 vs si-sc. Figure S5. Intravitreal injection of a GSK3β inhibitor TWS119 reduces retinal tau hyperphosphorylation and synapse loss in HFD-fed mice. (a-c) Immunostaining. *P < 0.05 and **P < 0.01 vs contralateral eye injected with vehicle. Figure S6. Intravitreal delivery of si-GSK3β protects RGCs from HFD-induced vision and synapse loss. (a) Representative VEP. (b) Knock-down efficiency of targeted si-GSK3β. (c-e) Representative immunostaining. n = 4 eyes per group. **P < 0.01 vs si-sc. Figure S7. Inhibition of GSK3β by TWS119 does not trigger disease phenotype under physiological conditions. (a) VEP. (b-c) Immunostaining. Figure S8. Glucolipotoxicity induces dysregulation of Akt/GSK3β signaling and tau hyperphosphorylation in primary RGCs. (a-b) Western blot. (c) Immunostaining. *P < 0.05 and **P < 0.01 vs control; #P < 0.05 and ##P < 0.01 vs HG + PA. Figure S9. Selected tau depletion by siRNA prevents RGCs from glucolipotoxicity-induced mitochondria dysfunction. (a) Western blot. (b) Immunostaining. *P < 0.05 and **P < 0.01 vs si-sc. Figure S10. Tau depletion or GSK3β inhibition restores HFD-impaired mitochondrail activity. (a) Western blot. (b-d) Retinal double immunostaini [file 13024_2018_295_MOESM1_ESM.zip › Suppl Fig.6-revision.pdf]

Suppl. Figure 7

a

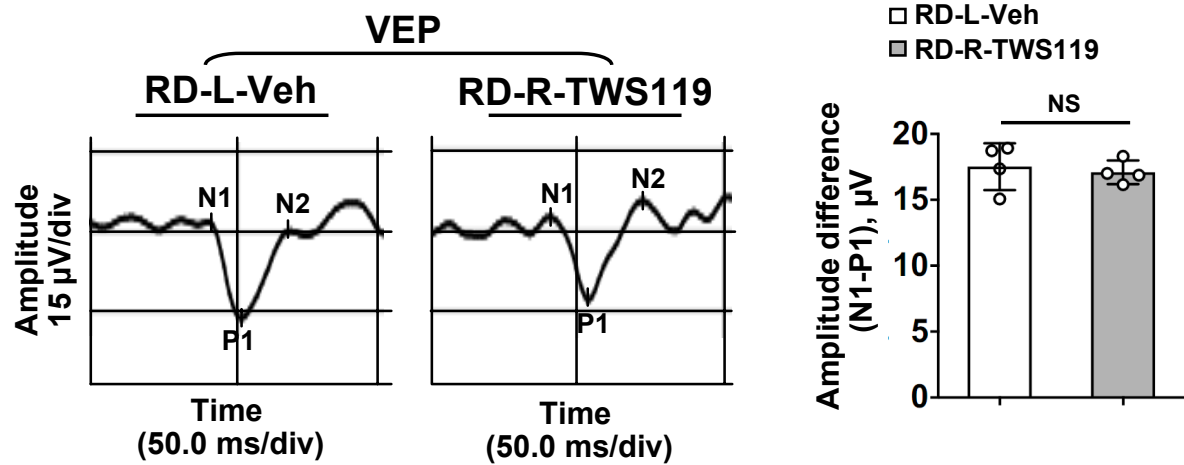

b

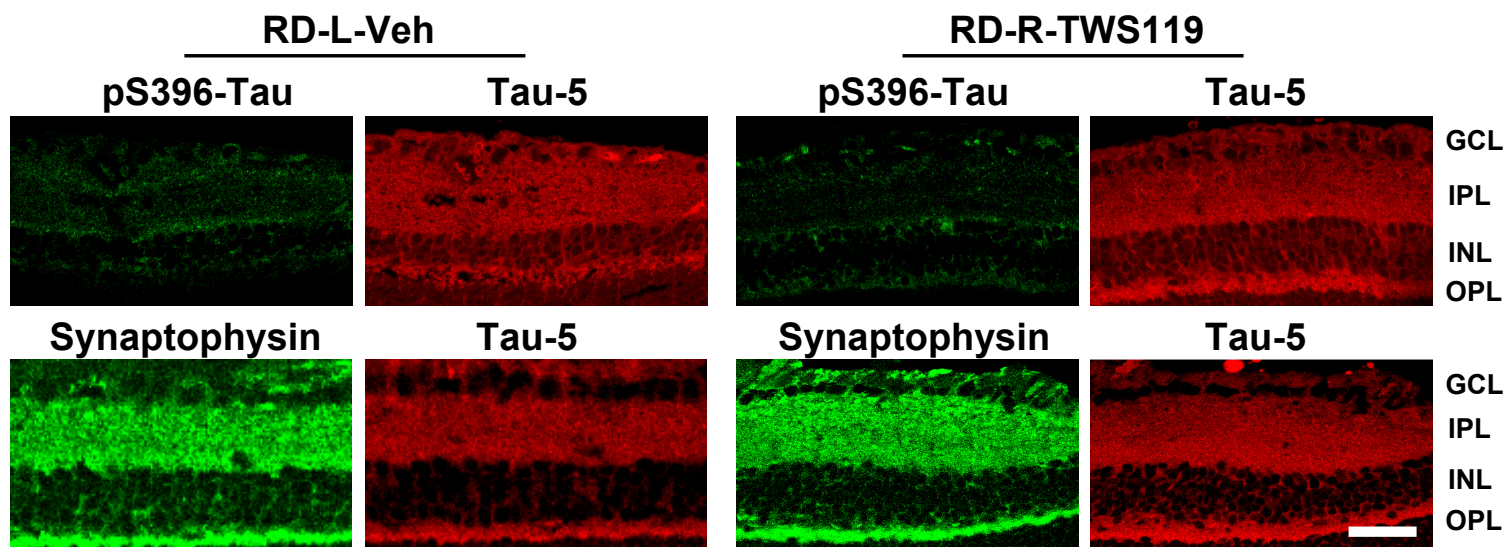

c

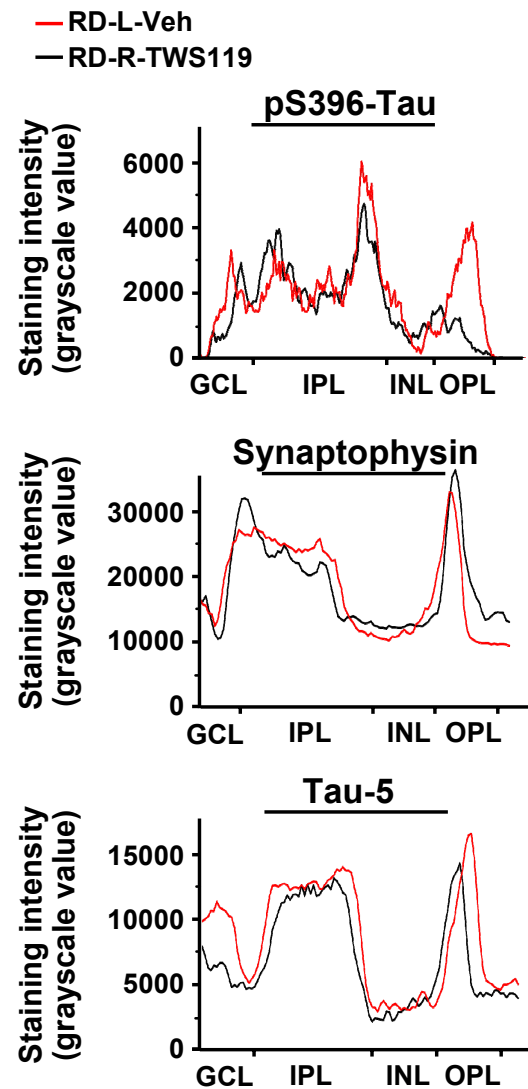

Supplement: Supplementary file 1 — Figure S1. Synapse loss occurs before apoptosis in RGCs from HFD-induced type 2 diabetic mice. (a-g) HFD-fed mice developed features of type 2 diabetes. (h-i) Western blot. (j) Evans Blue angiography. n = 6 (a-g) or = 4 (h-i) animals per group. *P < 0.05 and **P < 0.01 vs age-match RD controls. Figure S2. HFD promotes hyperphosphorylation of tau in neural retina and optic nerves. Double immunostaining in retina (a) or in RGCs axons (b). Figure S3. Knock-down of tau does not cause disease phenotype under physiological conditions. (a) Knock-down efficiency of a targeted si-Tau. n = 3 eyes per group. *P < 0.05 vs si-sc. (b-c) Immunostaining. Figure S4. Intravitreal delivery of si-Tau prevents RGCs from HFD-induced tau phosphorylation and synapse loss. (a-c) Immunostaining. *P < 0.05 and **P < 0.01 vs si-sc. Figure S5. Intravitreal injection of a GSK3β inhibitor TWS119 reduces retinal tau hyperphosphorylation and synapse loss in HFD-fed mice. (a-c) Immunostaining. *P < 0.05 and **P < 0.01 vs contralateral eye injected with vehicle. Figure S6. Intravitreal delivery of si-GSK3β protects RGCs from HFD-induced vision and synapse loss. (a) Representative VEP. (b) Knock-down efficiency of targeted si-GSK3β. (c-e) Representative immunostaining. n = 4 eyes per group. **P < 0.01 vs si-sc. Figure S7. Inhibition of GSK3β by TWS119 does not trigger disease phenotype under physiological conditions. (a) VEP. (b-c) Immunostaining. Figure S8. Glucolipotoxicity induces dysregulation of Akt/GSK3β signaling and tau hyperphosphorylation in primary RGCs. (a-b) Western blot. (c) Immunostaining. *P < 0.05 and **P < 0.01 vs control; #P < 0.05 and ##P < 0.01 vs HG + PA. Figure S9. Selected tau depletion by siRNA prevents RGCs from glucolipotoxicity-induced mitochondria dysfunction. (a) Western blot. (b) Immunostaining. *P < 0.05 and **P < 0.01 vs si-sc. Figure S10. Tau depletion or GSK3β inhibition restores HFD-impaired mitochondrail activity. (a) Western blot. (b-d) Retinal double immunostaini [file 13024_2018_295_MOESM1_ESM.zip › Suppl Fig.7-revision.pdf]

Suppl. Figure 8

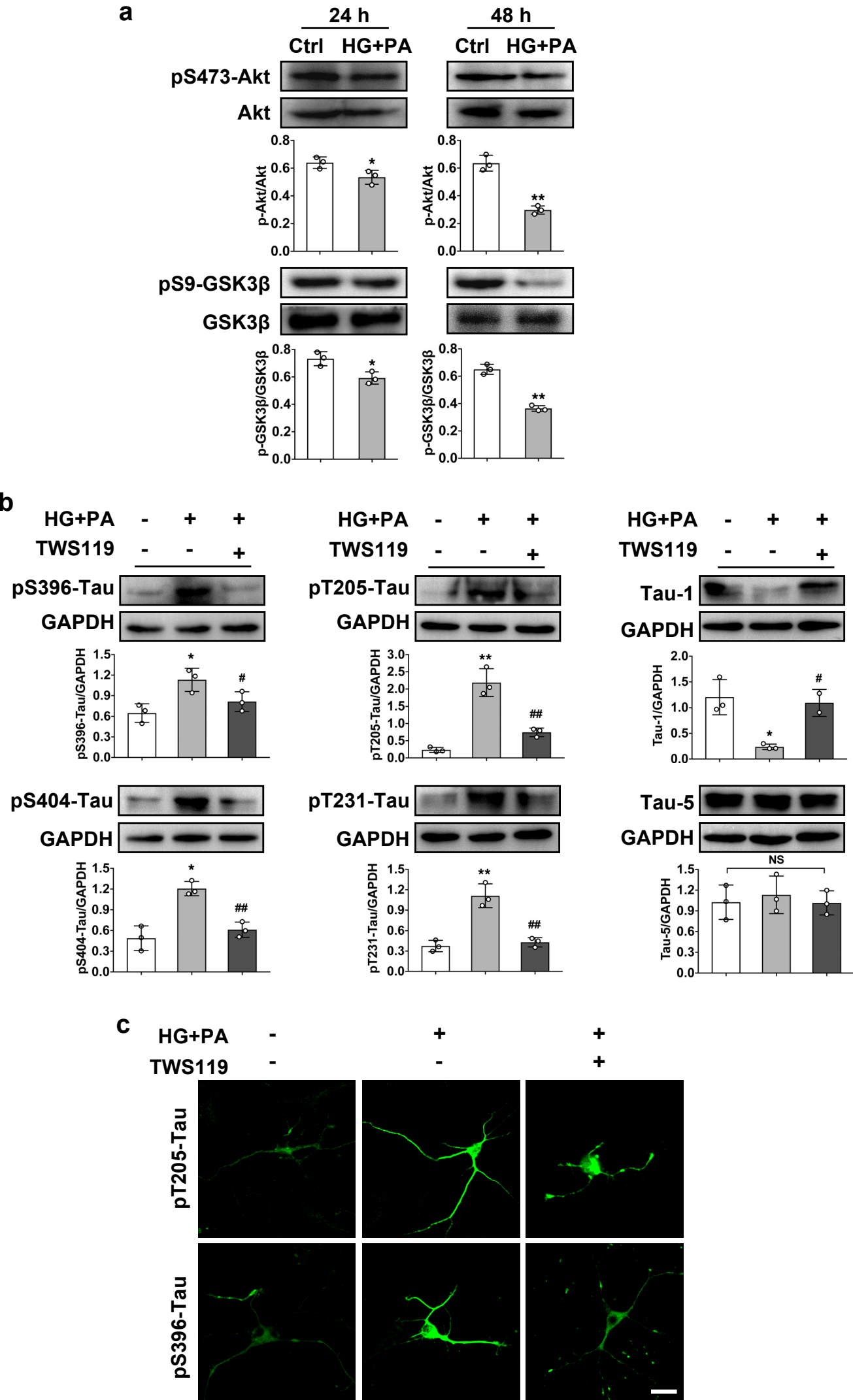

Supplement: Supplementary file 1 — Figure S1. Synapse loss occurs before apoptosis in RGCs from HFD-induced type 2 diabetic mice. (a-g) HFD-fed mice developed features of type 2 diabetes. (h-i) Western blot. (j) Evans Blue angiography. n = 6 (a-g) or = 4 (h-i) animals per group. *P < 0.05 and **P < 0.01 vs age-match RD controls. Figure S2. HFD promotes hyperphosphorylation of tau in neural retina and optic nerves. Double immunostaining in retina (a) or in RGCs axons (b). Figure S3. Knock-down of tau does not cause disease phenotype under physiological conditions. (a) Knock-down efficiency of a targeted si-Tau. n = 3 eyes per group. *P < 0.05 vs si-sc. (b-c) Immunostaining. Figure S4. Intravitreal delivery of si-Tau prevents RGCs from HFD-induced tau phosphorylation and synapse loss. (a-c) Immunostaining. *P < 0.05 and **P < 0.01 vs si-sc. Figure S5. Intravitreal injection of a GSK3β inhibitor TWS119 reduces retinal tau hyperphosphorylation and synapse loss in HFD-fed mice. (a-c) Immunostaining. *P < 0.05 and **P < 0.01 vs contralateral eye injected with vehicle. Figure S6. Intravitreal delivery of si-GSK3β protects RGCs from HFD-induced vision and synapse loss. (a) Representative VEP. (b) Knock-down efficiency of targeted si-GSK3β. (c-e) Representative immunostaining. n = 4 eyes per group. **P < 0.01 vs si-sc. Figure S7. Inhibition of GSK3β by TWS119 does not trigger disease phenotype under physiological conditions. (a) VEP. (b-c) Immunostaining. Figure S8. Glucolipotoxicity induces dysregulation of Akt/GSK3β signaling and tau hyperphosphorylation in primary RGCs. (a-b) Western blot. (c) Immunostaining. *P < 0.05 and **P < 0.01 vs control; #P < 0.05 and ##P < 0.01 vs HG + PA. Figure S9. Selected tau depletion by siRNA prevents RGCs from glucolipotoxicity-induced mitochondria dysfunction. (a) Western blot. (b) Immunostaining. *P < 0.05 and **P < 0.01 vs si-sc. Figure S10. Tau depletion or GSK3β inhibition restores HFD-impaired mitochondrail activity. (a) Western blot. (b-d) Retinal double immunostaini [file 13024_2018_295_MOESM1_ESM.zip › Suppl Fig.8-revision.pdf]

# Suppl. Figure 9

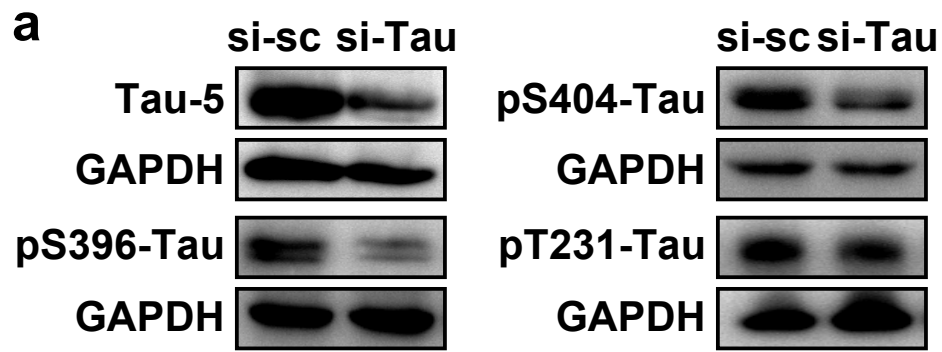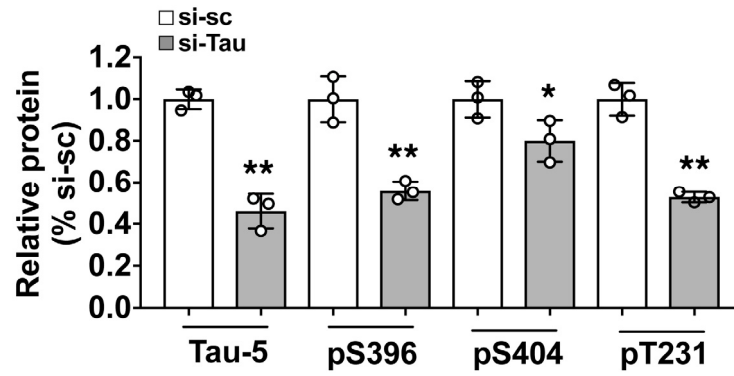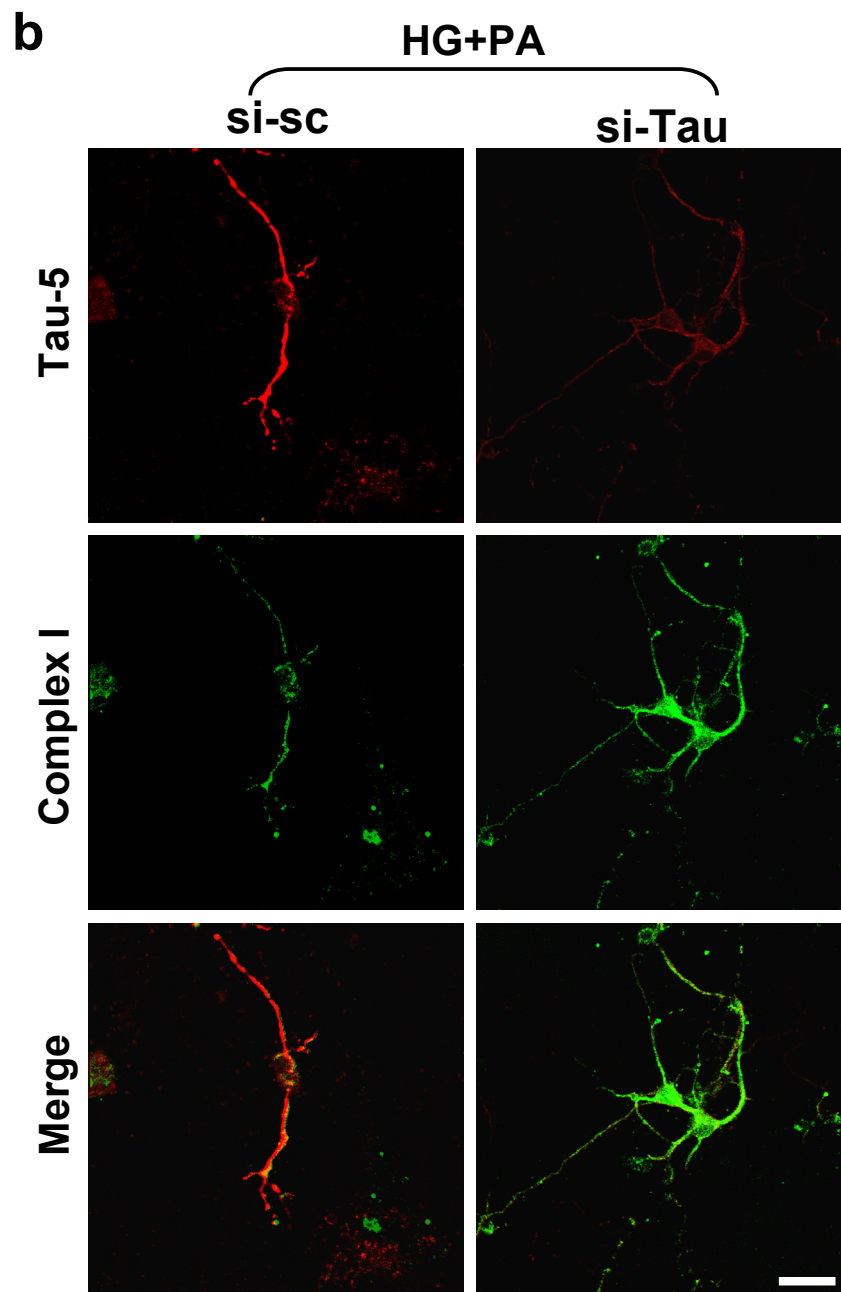

Supplement: Supplementary file 1 — Figure S1. Synapse loss occurs before apoptosis in RGCs from HFD-induced type 2 diabetic mice. (a-g) HFD-fed mice developed features of type 2 diabetes. (h-i) Western blot. (j) Evans Blue angiography. n = 6 (a-g) or = 4 (h-i) animals per group. *P < 0.05 and **P < 0.01 vs age-match RD controls. Figure S2. HFD promotes hyperphosphorylation of tau in neural retina and optic nerves. Double immunostaining in retina (a) or in RGCs axons (b). Figure S3. Knock-down of tau does not cause disease phenotype under physiological conditions. (a) Knock-down efficiency of a targeted si-Tau. n = 3 eyes per group. *P < 0.05 vs si-sc. (b-c) Immunostaining. Figure S4. Intravitreal delivery of si-Tau prevents RGCs from HFD-induced tau phosphorylation and synapse loss. (a-c) Immunostaining. *P < 0.05 and **P < 0.01 vs si-sc. Figure S5. Intravitreal injection of a GSK3β inhibitor TWS119 reduces retinal tau hyperphosphorylation and synapse loss in HFD-fed mice. (a-c) Immunostaining. *P < 0.05 and **P < 0.01 vs contralateral eye injected with vehicle. Figure S6. Intravitreal delivery of si-GSK3β protects RGCs from HFD-induced vision and synapse loss. (a) Representative VEP. (b) Knock-down efficiency of targeted si-GSK3β. (c-e) Representative immunostaining. n = 4 eyes per group. **P < 0.01 vs si-sc. Figure S7. Inhibition of GSK3β by TWS119 does not trigger disease phenotype under physiological conditions. (a) VEP. (b-c) Immunostaining. Figure S8. Glucolipotoxicity induces dysregulation of Akt/GSK3β signaling and tau hyperphosphorylation in primary RGCs. (a-b) Western blot. (c) Immunostaining. *P < 0.05 and **P < 0.01 vs control; #P < 0.05 and ##P < 0.01 vs HG + PA. Figure S9. Selected tau depletion by siRNA prevents RGCs from glucolipotoxicity-induced mitochondria dysfunction. (a) Western blot. (b) Immunostaining. *P < 0.05 and **P < 0.01 vs si-sc. Figure S10. Tau depletion or GSK3β inhibition restores HFD-impaired mitochondrail activity. (a) Western blot. (b-d) Retinal double immunostaini [file 13024_2018_295_MOESM1_ESM.zip › Suppl Fig.9-revision.pdf]
